# What Tests are Used to Assess the Physical Qualities of Male, Adolescent Rugby League Players? A Systematic Review of Testing Protocols and Reported Data Across Adolescent Age Groups

**DOI:** 10.1186/s40798-023-00650-z

**Published:** 2023-11-10

**Authors:** Michael A. Carron, Aaron T. Scanlan, Cody J. Power, Thomas M. Doering

**Affiliations:** 1 School of Health, Medical and Applied Sciences, Central Queensland University, Building 81, Bruce Highway, Rockhampton, QLD 4702 Australia; 2 Human Exercise and Training Laboratory, School of Health, Medical and Applied Sciences, Central Queensland University, Rockhampton, Australia

**Keywords:** Youth, Junior, Football, Fitness, Performance, Methods, Protocols, Physiology, Strength and conditioning

## Abstract

**Background:**

Understanding the physical qualities of male, adolescent rugby league players across age groups is essential for practitioners to manage long-term player development. However, there are many testing options available to assess these qualities, and differences in tests and testing protocols can profoundly influence the data obtained.

**Objectives:**

The aims of this systematic review were to: (1) identify the most frequently used tests to assess key physical qualities in male, adolescent rugby league players (12–19 years of age); (2) examine the testing protocols adopted in studies using these tests; and (3) synthesise the available data from studies using the most frequently used tests according to age group.

**Methods:**

A systematic search of five databases was conducted. For inclusion, studies were required to: (1) be original research that contained original data published in a peer-reviewed journal; (2) report data specifically for male, adolescent rugby league players; (3) report the age for the recruited participants to be between 12 and 19 years; (4) report data for any anthropometric quality and one other physical quality and identify the test(s) used to assess these qualities; and (5) be published in English with full-text availability. Weighted means and standard deviations were calculated for each physical quality for each age group arranged in 1-year intervals (i.e., 12, 13, 14, 15, 16, 17 and 18 years) across studies.

**Results:**

37 studies were included in this systematic review. The most frequently used tests to assess anthropometric qualities were body mass, standing height, and sum of four skinfold sites. The most frequently used tests to assess other physical qualities were the 10-m sprint (linear speed), 505 Agility Test (change-of-direction speed), Multistage Fitness Test (aerobic capacity), bench press and back squat one-repetition maximum tests (muscular strength), and medicine ball throw (muscular power). Weighted means calculated across studies generally demonstrated improvements in player qualities across subsequent age groups, except for skinfold thickness and aerobic capacity. However, weighted means could not be calculated for the countermovement jump.

**Conclusion:**

Our review identifies the most frequently used tests, but highlights variability in the testing protocols adopted. If these tests are used in future practice, we provide recommended protocols in accordance with industry standards for most tests. Finally, we provide age-specific references for frequently used tests that were implemented with consistent protocols.

***Clinical Trial Registration*** This study was conducted in accordance with the Preferred Reporting Items of Systematic Review and Meta-analysis guidelines and was registered with PROSPERO (ID: CRD42021267795).

**Supplementary Information:**

The online version contains supplementary material available at 10.1186/s40798-023-00650-z.

## Background

Rugby league is an intermittent, field-based team sport, requiring players to repeatedly complete high-intensity bouts, interspersed with activity performed at lower intensities [1]. Rugby league is played from amateur to professional levels worldwide [2] with the largest professional leagues hosted in Australia [3] and England [4]. In many countries, rugby league is also played competitively among male, adolescent players aged between 12 and 19 years [5]. Indeed, professional rugby league clubs and national governing bodies invest in youth academies and talent identification programmes [6] to identify and develop adolescent players with favourable physical qualities that may increase their likelihood of success in rugby league. In this regard, a review by Till and colleagues [4] showed that male, adolescent rugby league players possessing superior physical qualities (height, body mass, and sum of skinfolds, speed, change-of-direction [COD] speed, muscular strength, and muscular power) were more likely to progress from amateur to professional playing standards (whereby players are contracted to play). Given the importance of physical qualities in determining career outcomes of male, adolescent rugby league players, a comprehensive synthesis of data representing key qualities across different ages and playing levels (i.e., amateur, academy, and elite) is essential. These data may be used as benchmark standards, allowing rugby league coaching staff to best prepare their adolescent players for successful transition to higher playing levels. Unfortunately, despite the welcomed increase in participation and professionalisation among female rugby league players across all age groups, limited research has reported the physical qualities of female, adolescent rugby league players [7]. Therefore, this review will focus on male, adolescent rugby league players, but systematic synthesis of the literature on this topic is encouraged in female, adolescent rugby league players as the evidence base grows.

The demands of male, adolescent rugby league match-play necessitate players have well-developed physical qualities including high body mass and low sum of skinfolds [8], as well as high linear speed [9], COD speed [10], aerobic capacity [11], muscular strength [12], and muscular power [13], relative to their age. Accordingly, the physical qualities of male, adolescent rugby league players reported in the literature have been compiled in previous reviews [1, 4, 14]. For example, Till and colleagues [4] conducted the most comprehensive review to date, synthesising the reported values for selected physical qualities (i.e., height, body mass, and sum of skinfolds from 12 studies, muscular strength from 4 studies, and muscular power, linear speed, COD speed, and aerobic capacity from 11 studies) in elite male, adolescent rugby league players (13–20 years of age). While this previous review [4] offers useful insight, there is a need for an updated review for several reasons. Firstly, the previous review [4] only included studies examining “elite”, adolescent, male rugby league players, defined as those “who were selected for a national governing body talent identification and development programme or were members of a professional rugby league club academy programme”. Consequently, the physical qualities of male, adolescent rugby league players at non-elite playing levels, including those playing at amateur and school levels, remains to be synthesised. Secondly, the data representing physical qualities reported in the previous review [4] were not differentiated according to the test protocols implemented across studies [15], with detailed accounts of the testing protocols used to assess each quality being omitted. In this regard, identifying the most frequently used tests and testing protocols in the literature may assist in establishing future testing recommendations for male, adolescent rugby league players.

Recognising the value of identifying frequently used tests in the literature, Chiwaridzo and colleagues [16] conducted a systematic review on this topic in 2017, but several of the inclusion criteria used limit the specificity of the findings to male, adolescent rugby league players. For example, the previous review [16] encompassed both rugby league (71% of studies) and rugby union (26% of studies) players, with some studies combining players from both sports (3% of studies). Secondly, male, adult players were included in the previous review [16], who possess varied physical qualities compared to male, adolescent rugby league players [17] and may have access to varied facilities and equipment; thus older rugby league players may require, or have used different, testing protocols compared to younger players. Thirdly, a thorough evaluation of the testing protocols adopted when implementing each test was not provided in the previous review [16], which is important given testing protocol variations can impact the data obtained [15]. Therefore, identifying the most frequently used tests and testing protocols to measure physical qualities in male, adolescent rugby league players will enable future synthesis of similar data for comparison according to age group and playing level. Furthermore, identifying protocol discrepancies among the literature for specific tests may assist in recommending how they should be implemented in the future.

Therefore, the aims of this systematic review are to: (1) identify the most frequently used tests to assess key physical qualities in male, adolescent rugby league players (12–19 years of age); (2) examine the testing protocols adopted in studies using these tests; and (3) synthesise the available data from studies using the most frequently used tests according to age group.

## Methods

### Design and Search Strategy

This systematic review was conducted in accordance with the Preferred Reporting Items of Systematic Reviews and Meta-analyses (PRISMA) guidelines and was registered with PROSPERO (ID: CRD42021267795). Five databases were searched (PubMed, MEDLINE, Web of Science, Scopus, and SPORTDiscus) on 1 July 2022. A search strategy containing 33 keywords was employed with keywords divided into three levels, each linked by the Boolean operator ‘AND’. Keywords within each level were linked by the Boolean operator ‘OR’ (Additional file 1: Table A). All search results were exported from each database and imported into reference management software (EndNote, version X9.3.3; Clarivate Analytics, Microsoft Corp, Redmond, MA).

### Study Selection and Inclusion Criteria

Duplicate studies retrieved across multiple databases were removed within the reference management software. To be included in this systematic review, studies were required to: (1) be original research that contained original data (i.e., not previously reported in another study) published in a peer-reviewed journal; (2) report data specifically for male, adolescent rugby league players; (3) report the mean age for the recruited participants to be between 12 and 19 years; (4) report data for any anthropometric quality and one other physical quality and identify the test(s) used to assess these qualities; and (5) be published in English with full-text availability.

Studies were excluded if they: (1) reported previously published data, or were a narrative review, systematic review, or meta-analysis; (2) reported the age of the player sample outside of the range of 12–19 years; (3) included only female players or did not differentiate data according to sex if examining female and male players together; (4) did not report data for at least one anthropometric quality and at least one other physical quality concurrently, or provide these data on request via email communication if data were not clearly reported in the published version; (5) did not identify the test(s) used to assess the included physical qualities; or (6) adopted a longitudinal observational or experimental study design and did not report baseline data.

Observational and experimental studies were included in this systematic review, but baseline data (i.e., prior to the longitudinal monitoring period or implementation of any intervention) for the reported physical qualities were extracted to avoid any confounding influence of time or intervention. Two reviewers (MC and CP) independently screened titles and abstracts of all studies retrieved from the initial search, with all conflicts discussed. If conflicts could not be resolved through discussion, a third reviewer (AS) was consulted to provide a consensus decision (*n* = 1). Two reviewers (MC and CP) subsequently examined all full-text versions for final eligibility with all conflicts resolved via discussion.

### Assessment of Reporting Quality

An assessment of study quality was completed on each included study using a modified Downs and Black checklist [18]. The modified Downs and Black checklist has been implemented in systematic reviews quantifying demands and match metrics of rugby league players [19, 20], and is a valid risk-of-bias tool for observational studies [21]. In the modified Downs and Black checklist, a score of 11 is the highest quality score achievable (Additional file 2: Table B); however, one question (question 9) was not applicable to this review and therefore a score of 10 was the highest quality score achievable. Two reviewers (MC and CP) independently conducted the risk of bias and quality assessment, with three discrepancies arising between reviewers and resolved via discussion.

### Data Extraction

Data from all included studies were extracted into a customised spreadsheet (Microsoft Excel, version 16.54; Microsoft Corp, Redmond, MA). Data were extracted from each study by the lead author (MC) and verified by another author for accuracy (CP). Extracted data included author names, year of publication, sample size, mean age of player sample, any sub-group reported (i.e., playing level categorised into amateur, academy, and elite levels, and positional groups as stipulated in the study), tests conducted to assess any physical quality, the data (i.e., mean ± standard deviation) reported for each test, and protocols specific to each test. Where confidence intervals were reported (*n* = 4) [1, 22–24], standard deviations were calculated using these data according to the Cochrane Handbook for Systematic Reviews of Interventions (version 5.1.0) for consistency in reporting.

### Categorisation and Presentation of Findings

Data extracted from each included study were first categorised as physical qualities including anthropometric (height, body mass, and sum of skinfolds), linear speed, COD speed, aerobic capacity, muscular strength, and muscular power. Further, the total number of studies using each test and the total cumulative sample size of players assessed using each test were reported.

For data to be reported in this review and to consolidate our reporting of the most *frequently* used tests in the literature, permitting reasonable comparison of physical qualities, a test must have: (1) been used in a minimum of three studies; and (2) assessed at least 5% of the total cumulative sample reported in the literature for that quality. For example, if 6000 players were assessed for linear speed across all included studies, a test must have been used in at least three studies, and cumulatively assessed at least 300 players to be reported. While *all* tests identified from *all* included studies are reported in Table 1, only data from *frequently* used tests are reported in this review.

**Table 1:** Frequency of tests used to measure physical qualities in male, adolescent rugby league players reported within the literature

Quality	Test	Number of studies (and specific reference) that utilise test (*n*)	Percentage of studies that utilise test (%)	Total sample assessed with test (*n*)	Percentage of sample assessed with test (%)
*Anthropometric*					
(37 studies, *N* = 6083)	Standing height (cm)	33 [8, 13, 14, 22–24, 38, 39, 42, 45, 47–52, 56, 59–66, 72, 84, 86, 95–97, 102–105]	89	5783	95
	Seated height (cm)	7 [8, 39, 46, 50, 60, 95, 102]	19	724	12
	Body mass (kg)	37 [1, 8, 13, 14, 22–24, 38, 39, 42, 45–52, 56, 59–66, 72, 84, 86, 95, 96, 102–105]	100	6083	100
	Σ4 sites skinfold sites (mm)	14 [8, 22, 39, 45, 48, 50, 51, 59, 61, 95–97, 102, 103]	38	4042	66
	Σ6 sites skinfold sites (mm)	1 [46]	3	13	< 1
	Σ7 sites skinfold sites (mm)	7 [14, 23, 38, 42, 47, 49, 60]	19	438	7
	Σ8 sites skinfold sites (mm)	1 [65]	3	214	4
	Σ9 sites skinfold sites (mm)	1 [86]	3	65	1
	Body fat (%) by estimation equation [76, 77]	1 [46]	3	129	2
	Lean mass by estimation equation [77]	1 [46]	3	13	< 1
*Linear speed*					
(33 studies, *N* = 5789)	10-m sprint time (s)	31 [1, 8, 13, 14, 22–24, 38, 39, 42, 45, 47–51, 56, 59–66, 95–97, 102–104]	94	5415	94
	20-m sprint time (s)	29 [1, 8, 13, 14, 22, 23, 39, 42, 45–51, 56, 59, 61–66, 95–97, 102–104]	88	5482	95
	30-m sprint time (s)	11 [8, 39, 45, 50, 51, 56, 63, 72, 95, 102, 104]	33	3137	54
	40-m sprint time (s)	12 [1, 13, 14, 22–24, 47–49, 56, 60, 63]	36	1135	20
	60-m sprint time (s)	7 [8, 39, 45, 50, 51, 95, 102]	21	1254	22
	Velocity across 0–10 m (m s^−1^)	1 [14]	3	88	2
	Velocity across 10–20 m (m s^−1^)	1 [14]	3	88	2
	Velocity across 20–40 m (m s^−1^)	1 [14]	3	88	2
	10-m sprint momentum (kg s^−1^)	1 [103]	3	55	1
*Change of direction speed*					
(19, studies *N* = 3765)	505 Agility Test (s)	12 [8, 14, 24, 38, 39, 45, 47, 48, 50, 51, 95, 102]	75	3197	89
	L-test (s)	4 [22, 23, 49, 72]	25	505	13
	T-test (s)	2 [8, 50]	13	283	8
	Change of direction test (s)	1 [42]	6	729	19
	Illinois test (s)	1 [1]	6	159	4
*Aerobic capacity*					
(28 Studies, *N* = 5636)	Multistage fitness test (predicted $\dot{\mathrm{V}}$O_2max_)	19 [1, 8, 14, 22–24, 39, 45–52, 60, 95, 96, 102]	71	4194	74
	Yo–Yo intermittent Recovery Test 1 (m)	9 [42, 59, 61, 62, 66, 72, 97, 103, 104]	32	1442	26
	Continuous running ability (s)	1 [72]	4	63	1
*Muscular strength*					
(9 studies, *N* = 691)	Bench press 1RM (kg)	9 [13, 59, 61, 84, 86, 96, 97, 103, 105]	100	691	100
	Back squat 1RM (kg)	7 [13, 59, 61, 84, 96, 97, 103]	78	639	92
	Prone row 1RM (kg)	5 [59, 61, 96, 97, 103]	56	228	33
*Muscular power*					
(31 studies, *N* = 5797)	Countermovement jump (cm)	31 [1, 8, 13, 14, 22–24, 38, 39, 42, 45–52, 59–62, 64–66, 72, 95–97, 102, 103]	100	5797	100
	Medicine ball throw (m)	10 [8, 39, 42, 45, 50, 51, 62, 66, 95, 102]	32	3643	63
	Bench throw with 20 kg (W)	1 [105]	3	95	2
	Jump squat with 20 kg (W)	1 [105]	3	95	2
	Peak lower limb power (W)	1 [72]	3	174	3

Data included in this review were then organised by physical quality. All physical qualities were reported in separate tables. Within these tables, studies are ordered based on the mean chronological age of the player sample reported and assigned to the appropriate age group spanning 12–19 years in 1-year intervals. In studies reporting data for multiple age categories (i.e., 14, 15, and 16 years), data for those respective groups were extracted, and reported independently in our review. Data and sample size are reported according to the positional subgroups reported in each study. Furthermore, we categorised the playing levels of study participants as: (1) amateur (a voluntary non-contractual player, competing for a school or club); (2) academy (contracted to participate as part of an institution/club); and (3) elite (contracted to a professional club and/or selected to play at a regional or higher representative level). These definitions were developed in accordance with a recent framework [25] given the literature included in this review reported playing levels using several different terms such as “sub-elite” and “academy”.

For the most frequently used tests, the procedural description was checked against test-specific criteria the authors deemed essential for test replication in practice that may also impact the data obtained. Specifically, criterion protocols crosschecked in every study using the 10-m sprint were: (1) starting distance behind the line; (2) number of trials conducted and whether the mean time (s) or best time (s) across trials was reported; and (3) equipment used to assess sprint time. Criterion protocols crosschecked in every study using the 505 Agility Test were: (1) number of trials and whether the mean time (s) or best time (s) across trials was reported; and (2) equipment used to measure COD performance time. There were no specific criterion protocols crosschecked for studies using the Multistage Fitness Test (MSFT), as studies consistently reported protocols stipulated by Ramsbottom [26]. Criterion protocols crosschecked in every study using the one-repetition maximum (1RM) (for squat and bench press) were: (1) number of attempts; (2) rest duration between each attempt; and (3) equipment used to measure 1RM. Criterion protocols crosschecked in every study using the countermovement jump (CMJ) were: (1) whether an arm swing was permitted; (2) number of trials and whether the mean jump height (cm) or best jump height (cm) across trials was reported; and (3) equipment used to measure jump height. Criterion protocols crosschecked in every study using the medicine ball throw (MBT) were: (1) the position in which the player performed the throw; (2) mass of the medicine ball; and (3) number of trials and whether the mean throw distance (m) or best throw distance (m) across trials was reported.

Finally, to understand the progression of player qualities across age groups, data for each included test were combined to provide a mean value for each frequently used test in each age group. Importantly, only studies that used the most frequently adopted protocols for the given test were included in this calculation; for example, where a study used a 10-m sprint test, the reported data would be used to calculate the weighted mean and standard deviation if implemented using electronic light gates to record the best time across three trials, with players starting 0.5 m from the first light gate. To provide mean values, all data for a given test within an age group were weighted according to sample size (e.g., [mean height (*A*) × sample (*A*)] + [mean height (*B*) × sample (*B*)]/[total sample (*A* + *B*)]). This method was also applied to standard deviation values. Given the low number of studies examining amateur and elite players, and inconsistencies in reporting playing positions across studies, normative mean values were not able to be calculated according to playing level or positional groups. Furthermore, due to a lack of methodological consistency in protocols of the CMJ across the studies included in this review, the calculation of a weighted mean for the CMJ test was precluded.

## Results

### Identification and Selection of Articles

**Fig. 1:**
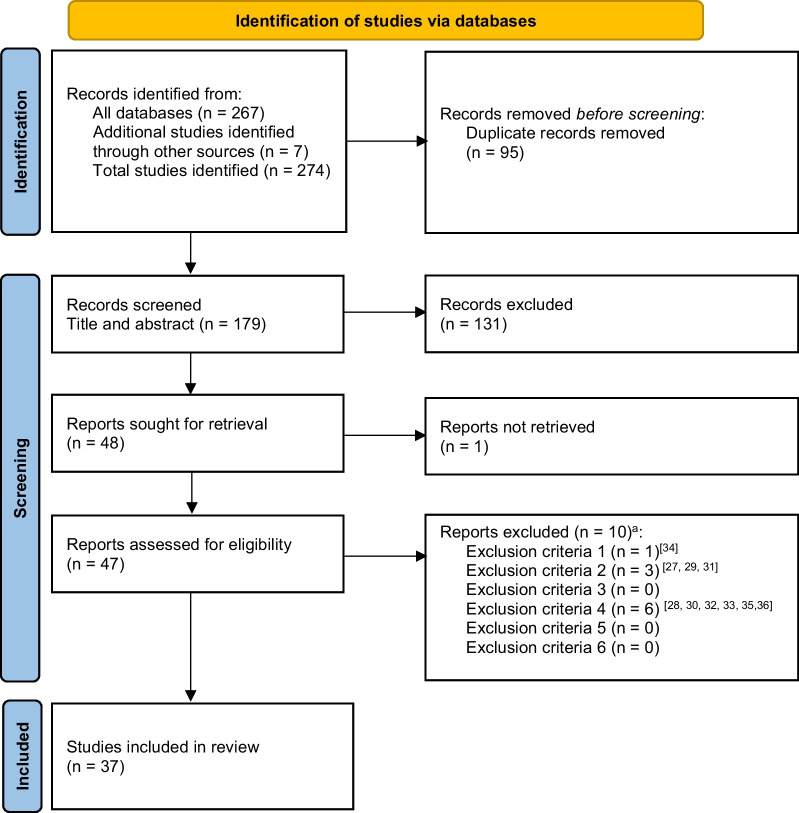
Selection process of eligible studies for synthesis in this review. *Notes*: ^a^Reports excluded according to exclusion criteria: (1) reported previously published data or were a narrative review, systematic review, or meta-analysis; (2) reported mean age of the sample outside the specified age range of 12–19 years; (3) included only female players or did not differentiate data according to sex if examining female and male players together; (4) did not report data for at least one physical characteristic and at least one physiological characteristic concurrently, or did not provide these data on request via email communication if data were not clearly reported in the published study; (5) did not identify the test(s) used to assess the included physical; (6) adopted a longitudinal observational or experimental design and did not report baseline data

Searches across databases identified a total of 267 relevant studies, and a further seven studies were identified via manual searches of reference lists and deemed eligible for inclusion (*n* = 274). Ninety-five duplicate studies were removed, and the title and abstract of the remaining 179 studies were screened by two independent reviewers (MC and CP). A total of 131 studies did not meet the inclusion criteria and the remaining 48 studies were eligible for full-text review. The full-text version of one study could not be retrieved, and 10 studies [27–36] were excluded in the full-text screening process. As such, 37 studies were included in this systematic review (Fig. 1).

### Assessment of Reporting Quality

Study quality scores ranged from 6 to 10 for the 10 items assessed in the modified Downs and Black checklist. No studies were excluded based on risk of bias and methodological quality, with the mean score across included studies being 7.7 ± 1.2 out of 10 (Additional file 3: Table C).

### Frequency of Test Use

Table 1 shows the frequency of use for each individual test to assess physical qualities in male, adolescent rugby league players. A total of 37 studies examined the physical qualities of male, adolescent rugby league players, employing 35 different tests (Table 1).

A total of 37 studies examined anthropometric qualities using 10 different tests to assess standing height, body mass and skinfold thickness, but five tests were not reported in our dataset due to limited use and inadequate sample size; therefore, five frequently used tests examining anthropometric qualities are reported in this review from 37 studies. Furthermore, 33 studies examined linear speed using nine different tests, but four tests were not reported in our dataset due to limited use and inadequate sample size; therefore, five frequently used tests examining linear speed are reported in this review from 33 studies. Nineteen studies examined COD speed using five different tests, but three tests were not reported in our dataset due to limited use; therefore, two frequently used tests examining COD speed are reported in this review from 16 studies. Twenty-eight studies assessed aerobic capacity using three different tests, but one test was not reported in our dataset due to limited use and inadequate sample size; therefore, two frequently used tests examining aerobic capacity are reported in this review from 28 studies. Nine studies assessed muscular strength using three different tests, and therefore, all three tests examining muscular strength are reported in this review from all nine studies. Thirty-one studies assessed muscular power using five different tests, but three tests were not reported in our dataset due to limited use and inadequate sample size; therefore, two frequently used tests examining muscular power are reported in this review from 31 studies.

### Anthropometric Qualities

All 37 studies examined anthropometric qualities (Table 2), with measures of standing height assessed via stadiometer, body mass assessed via electronic scales, and skinfold thickness assessed via Σ4 skinfold thickness using Harpenden callipers the most frequently assessed qualities and tests. Most studies examined 17-year-old players (21 studies, 57%) with the least frequently studied age group being 12-year-old players (one study, 3%). A total sample of 6083 players were included across all studies examining anthropometric qualities. Across all ages, studies most frequently assessed academy players (35 studies, 95%), followed by elite (six studies, 16%), and amateur players (four studies, 11%). Seven studies (19%) reported data for players competing at multiple playing levels. Most studies (31 studies, 84%) did not report data according to playing position and grouped data for all players collectively.

**Table 2:** Anthropometric qualites reported in male, adolescent rugby league players according to age group, playing level, and playing position

Study	Playing level	Position	Sample size (*n*)	Age (years)	Height (cm)	Seated height (cm)	Body mass (kg)	Sum of skinfolds (mm)	Equipment		
									Stature	Body mass	Skinfolds
*12 years of age*											
Gabbett et al. [1]^a^	Academy	Forwards	13	12.5 ± 0.5	–	–	57.0 ± 13.2	–	–	Electronic Scales	–
		Backs	14	12.3 ± 0.5	–	–	44.8 ± 5.7	–			
*13 years of age*											
Gabbett et al. [48]	Academy	All	53	13.2 ± 0.6	161.5 ± 10.0		54.0 ± 15.0	Σ4: 34.2 ± 15.8	Stadiometer	Electronic scales	Harpenden
Gabbett et al. [1]^a^	Academy	Forwards	7	13.5 ± 0.5	–	–	67.7 ± 11.0	–	–	Electronic Scales	–
		Backs	10	13.7 ± 0.4	–	–	52.1 ± 6.6	–			
Till et al. [51]	Academy	All	221	13.6 ± 0.3	169.6 ± 7.7	–	62.2 ± 10.4	Σ4: 36.6 ± 14.9	Stadiometer	Electronic Scales	Harpenden
Till et al. [95]	Academy	All	NR	13.8 ± 0.1	172.8 ± 6.4	87.0 ± 3.7	65.1 ± 9.6	Σ4: 36.9 ± 17.0	Stadiometer	Electronic Scales	Harpenden
Till et al. [45]	Elite^b^	NR	255	13.6 ± 0.3	169.6 ± 8.4	–	62.4 ± 11.4	Σ4: 38.6 ± 16.4	Stadiometer	Electronic Scales	Harpenden
	Elite^c^		130	13.7 ± 0.2	171.0 ± 7.1	–	63.7 ± 9.0	Σ4: 34.9 ± 12.3			
Till et al. [8]	Amateur	All	249	13.6 ± 0.6	174.2 ± 7.1	88.9 ± 4.0	70.7 ± 13.5	Σ4: 41.6 ± 18.2	Stadiometer	Electronic Scales	Harpenden
	Academy	All	261	13.6 ± 0.6	175.2 ± 6.8	89.1 ± 4.0	70.9 ± 11.1	Σ4: 38.4 ± 15.5			
	Elite	All	70	13.8 ± 0.7	174.1 ± 9.7	87.8 ± 5.8	67.1 ± 12.8	Σ4: 33.4 ± 9.8			
Till et al. [50]	Amateur	All	NR	13.6 ± 0.2	171.4 ± 6.7	87.2 ± 4.4	65.4 ± 12.4	Σ4: 41.4 ± 20.3	Stadiometer	Electronic Scales	Harpenden
	Academy	All		13.7 ± 0.1	170.5 ± 4.6	86.6 ± 3.5	62.6 ± 7.6	Σ4: 35.8 ± 14.8			
	Elite	All		13.4 ± 0.3	170.6 ± 7.9	85.6 ± 4.2	63.0 ± 11.4	Σ4: 33.4 ± 13.7			
Till et al. [102]	Academy	All	NR	13.6 ± 0.2	171.2 ± 7.0	86.4 ± 4.1	63.9 ± 9.8	Σ4: 36.2 ± 15.0	Stadiometer	Electronic Scales	Harpenden
		Outside backs	NR	13.7 ± 0.2	171.5 ± 6.8	86.2 ± 4.0	60.3 ± 4.0	Σ4: 26.9 ± 5.8			
		Pivots	NR	13.5 ± 0.3	165.0 ± 5.6	83.7 ± 4.3	55.5 ± 7.1	Σ4: 31.9 ± 11.0			
		Props	NR	13.6 ± 0.2	174.0 ± 7.5	87.8 ± 4.0	75.0 ± 8.8	Σ4: 52.3 ± 18.6			
		Backs	NR	13.7 ± 0.2	174.2 ± 4.6	87.8 ± 3.1	67.2 ± 6.7	Σ4: 39.0 ± 12.1			
Till et al. [39]	Amateur	All	50	13.6 ± 0.3	169.0 ± 7.5	85.8 ± 4.1	61.2 ± 11.6	Σ4: 36.9 ± 17.0	Stadiometer	Electronic Scales	Harpenden
	Academy	All	32	13.6 ± 0.2	170.5 ± 5.9	86.6 ± 4.2	61.5 ± 7.7	Σ4: 33.3 ± 13.2			
	Elite	All	13	13.6 ± 0.3	166.8 ± 10.4	83.5 ± 5.5	57.2 ± 12.2	Σ4: 32.0 ± 6.8			
*14 years of age*											
Gabbett et al. [47]	Academy	All	14	14.1 ± 0.2	169.5 ± 2.1		65.9 ± 2.7	Σ7: 73.3 ± 8.4	Stadiometer	Electronic scales	Harpenden
Gabbett et al. [1]^a^	Academy	Forwards	11	14.5 ± 0.5	–	–	76.5 ± 9.7	–	–	Electronic Scales	–
		Backs	12	14.6 ± 0.5	–	–	62.1 ± 6.9	–			
Gabbett et al. [24]	Academy	All	23	14.5 ± 0.5	173.0 ± 9.3	–	69.0 ± 11.2	–	NR	NR	–
	Elite	All	36	14.3 ± 0.9	173.0 ± 8.9	–	73.9 ± 16.2	–			
Alonso-Aubin et al. [84]	Amateur	All	46	14.5 ± 1.3	165.0 ± 0.1	–	58.1 ± 13.0	–			
Till et al. [51]	Academy	All	240	14.6 ± 0.3	174.7 ± 6.4	–	70.0 ± 11.1	Σ4: 39.1 ± 16.8	Stadiometer	Electronic Scales	Harpenden
Till et al. [45]	Elite^b^	All	309	14.6 ± 0.3	175.0 ± 6.5	–	70.2 ± 10.8	Σ4: 40.1 ± 17.1	Stadiometer	Electronic Scales	Harpenden
	Elite^c^	All	86	14.6 ± 0.3	175.3 ± 6.5	–	71.1 ± 9.3	Σ4: 35.8 ± 12.2			
Till et al. [50]	Amateur	All	NR	14.6 ± 0.2	175.0 ± 6.1	89.2 ± 4.5	70.1 ± 12.3	Σ4: 44.5 ± 17.4	Stadiometer	Electronic Scales	Harpenden
	Academy	All	NR	14.7 ± 0.1	174.7 ± 4.7	89.3 ± 2.8	69.5 ± 9.0	Σ4: 35.4 ± 16.2			
	Elite	All	NR	14.4 ± 0.3	176.6 ± 5.8	89.3 ± 3.2	71.6 ± 10.6	Σ4: 37.4 ± 14.3			
Till et al. [102]	Academy	All	NR	14.6 ± 0.2	175.7 ± 6.2	89.3 ± 3.4	71.1 ± 9.6	Σ4: 39.3 ± 15.0	Stadiometer	Electronic Scales	Harpenden
		Outside backs	NR	14.7 ± 0.2	175.7 ± 6.4	89.0 ± 3.3	67.9 ± 5.3	Σ4: 32.2 ± 10.7			
		Pivots	NR	14.5 ± 0.3	171.1 ± 4.6	87.4 ± 3.2	63.2 ± 7.9	Σ4: 34.4 ± 13.9			
		Props	NR	14.6 ± 0.2	178.1 ± 6.9	90.4 ± 3.5	81.5 ± 8.6	Σ4: 53.2 ± 13.6			
		Backs	NR	14.7 ± 0.2	178.3 ± 4.0	90.7 ± 2.6	74.3 ± 7.4	Σ4: 41.5 ± 14.3			
Till et al. [39]	Amateur	All	92	14.6 ± 0.3	174.9 ± 6.2	89.0 ± 3.3	71.1 ± 11.8	Σ4: 41.2 ± 18.7	Stadiometer	Electronic Scales	Harpenden
	Academy	All	85	14.6 ± 0.3	174.8 ± 6.3	88.6 ± 3.7	70.0 ± 10.7	Σ4: 37.4 ± 13.8			
	Elite	All	18	14.5 ± 0.3	171.9 ± 9.0	86.0 ± 5.4	61.7 ± 9.2	Σ4: 28.5 ± 9.2			
Till et al. [59]	Academy	All	31	NR	–	–	55.0 ± 12.3	Σ4: 32.1 ± 8.6	–	Electronic Scales	Harpenden
Till et al. [95]	Academy	All	NR	14.8 ± 0.1	176.8 ± 6.1	89.7 ± 3.3	71.8 ± 9.2	Σ4: 38.9 ± 15.3	Stadiometer	Electronic Scales	Harpenden
*15 years of age*											
Dobbin et al. [66]	Academy	All	235	15.1 ± 0.8	172.6 ± 6.9	–	73.6 ± 10.6	–	Stadiometer	Electronic Scales	–
		Wingers	–	15.1 ± 0.8	174.6 ± 5.9	–	69.3 ± 9.7	–			
		Centre	–	15.1 ± 0.8	177.1 ± 5.2	–	72.6 ± 7.5	–			
		Halves	–	15.1 ± 0.9	172.9 ± 8.4	–	66.4 ± 8.1	–			
		Hookers	–	15.1 ± 0.1	171.6 ± 7.2	–	68.7 ± 10.5	–			
		Props	–	15.1 ± 0.1	178.4 ± 5.1	–	85.3 ± 9.4	–			
		Back row	–	15.1 ± 0.1	179.2 ± 6.2	–	77.3 ± 8.3	–			
Waldron et al. [52]	Academy	All	21	15.1 ± 0.3	178.6 ± 3.9	–	79.6 ± 9.1	–	NR	NR	
Gabbett et al. [48]	Academy	All	20	15.1 ± 0.6	171.6 ± 6.6	–	67.0 ± 17.7	Σ4: 31.1 ± 17.3	Stadiometer	Electronic scales	Harpenden
Waldron et al. [46]	Academy	All	13	15.1 ± 0.3	–	93.4 ± 2.3	81.9 ± 9.1	–	–	Electronic Scales	–
Fernandes et al. [86]	Academy	All	16	15.4 ± 0.5	–	–	79.7 ± 10.8	–	–	Electronic Scales	–
Gabbett et al. [1]^a^	Academy	Forwards	12	15.4 ± 0.9	–	–	75.5 ± 10.4	–	–	Electronic Scales	–
		Backs	9	15.4 ± 0.5	–	–	64.8 ± 16.0	–	–	–	–
Till et al. [51]	Academy	All	220	15.5 ± 0.3	177.8 ± 6.3	–	75.9 ± 10.4	Σ4: 41.2 ± 16.2	Stadiometer	Electronic Scales	Harpenden
Tredrea et al. [60]	Academy	All	49	15.5 ± 0.6	175.5 ± 6.7	89.9 ± 2.3	74.4 ± 13.4	Σ7: 76.9 ± 36.9	Stadiometer	Electronic scales	NR
	Elite	All	51	15.5 ± 0.5	177.1 ± 6.4	90.2 ± 2.5	77.3 ± 11.0	Σ7: 70.7 ± 6.4	–	–	–
Baker et al. [105]	Academy	All	13	15.5 ± 0.5	178.0 ± 4.4	–	83.2 ± 9.8	–	NR	NR	–
Till et al. [39]	Amateur	All	107	15.6 ± 0.3	177.1 ± 6.4	90.9 ± 3.6	76.1 ± 12.4	Σ4: 43.5 ± 17.0	Stadiometer	Electronic Scales	Harpenden
	Academy	All	144	15.6 ± 0.3	177.7 ± 6.3	90.7 ± 3.6	75.3 ± 10.4	Σ4: 40.4 ± 16.4	–	–	–
	Elite	All	39	15.5 ± 0.3	179.5 ± 5.8	91.4 ± 3.3	76.2 ± 8.5	Σ4: 37.3 ± 10.8	–	–	–
Till et al. [102]	Academy	All	NR	15.6 ± 0.2	178.6 ± 5.7	91.1 ± 3.1	77.6 ± 9.8	Σ4: 42.4 ± 16.0	Stadiometer	Electronic Scales	Harpenden
		Outside backs	NR	15.7 ± 0.2	178.3 ± 6.4	90.8 ± 3.1	73.4 ± 6.3	Σ4: 33.9 ± 8.9			
		Pivots	NR	15.5 ± 0.3	175.0 ± 4.1	89.5 ± 2.9	70.0 ± 6.3	Σ4: 35.6 ± 12.5			
		Props	NR	15.6 ± 0.2	180.2 ± 6.6	91.8 ± 3.6	88.5 ± 9.3	Σ4: 58.8 ± 16.5			
		Backs	NR	15.7 ± 0.2	180.7 ± 4.0	92.3 ± 2.3	80.7 ± 7.3	Σ4: 45.3 ± 14.9			
Gabbett et al. [24]	Academy	All	28	15.6 ± 0.6	176.0 ± 10.4	–	73.0 ± 12.3	–	NR	NR	–
	Elite	All	21	15.8 ± 0.3	177.0 ± 5.2	–	82.1 ± 10.9	–			
Till et al. [50]	Amateur	All	NR	15.6 ± 0.2	178.1 ± 5.0	91.3 ± 4.0	77.4 ± 11.4	Σ4: 46.2 ± 19.0	Stadiometer	Electronic Scales	Harpenden
	Academy	All	NR	15.7 ± 0.1	177.3 ± 5.0	91.0 ± 2.7	76.2 ± 10.4	Σ4: 42.3 ± 18.2			
	Elite	All	NR	15.4 ± 0.3	179.6 ± 4.2	91.4 ± 2.2	78.7 ± 10.3	Σ4: 36.8 ± 13.3			
Till et al. [45]	Elite^b^	All	306	15.6 ± 0.3	177.8 ± 6.3	–	75.8 ± 10.9	Σ4: 41.1 ± 15.8	Stadiometer	Electronic Scales	Harpenden
	Elite^c^	All	86	15.6 ± 0.3	178.3 ± 6.4	–	77.6 ± 9.7	Σ4: 39.9 ± 14.5			
Till et al. [61]	Academy	All	35	15.7 ± 0.2	176.7 ± 5.5	–	76.4 ± 8.4	Σ4: 35.0 ± 10.9	Stadiometer	Electronic Scales	Harpenden
Till et al. [95]	Academy	All	NR	15.8 ± 0.1	178.8 ± 6.1	91.1 ± 3.0	78.0 ± 10.0	Σ4: 42.7 ± 17.0	Stadiometer	Electronic Scales	Harpenden
Gabbett et al. [38]	Academy	All	13	15.9 ± 0.6	175.2 ± 6.9	–	72.3 ± 11.7	Σ7: 76.4 ± 28.1	Stadiometer	Electronic scales	Harpenden
Till et al. [96]	Academy	All	68	NR	175.7 ± 7.1	–	75.2 ± 11.1	Σ4: 37.2 ± 12.7	Stadiometer	Electronic Scales	Harpenden
		Forwards	37	NR	177.7 ± 5.4	–	80.9 ± 9.7	Σ4: 42.7 ± 14.1			
		Backs	31	NR	173.1 ± 8.2	–	68.4 ± 8.6	Σ4: 30.4 ± 5.9			
Till et al. [59]	Academy	All	75	NR	–	–	70.9 ± 11.1	Σ4: 38.2 ± 16.6	–	Electronic Scales	Harpenden
*16 years of age*											
Gabbett et al. [14]	Academy	All^d^	36	15.9 ± 0.6	176.0 ± 6.0	–	74.3 ± 13.4	Σ7: 75.1 ± 31.9	Stadiometer	Electronic scales	Harpenden
		Hit up forwards^d^	15.8 ± 0.7	176.7 ± 5.3	–	88.9 ± 7.2	Σ7: 113.6 ± 21.9				
		Adjustables^d^		15.7 ± 0.4	174.5 ± 3.3	–	68.0 ± 8.2	Σ7: 60.6 ± 7.9			
		Outside backs		16.1 ± 0.7	176.4 ± 7.4	–	69.2 ± 12.2	Σ7: 60.8 ± 25.7			
	Elite	All	28	16.0 ± 0.2	178.0 ± 5.9	–	77.5 ± 10.0	Σ7: 67.1 ± 14.8			
		Hit up forwards^d^	15.9 ± 0.4	180.9 ± 6.7	–	87.0 ± 11.1	Σ7: 80.2 ± 12.4				
		Adjustables		16.0 ± 0.2	175.5 ± 4.9	–	73.0 ± 6.2	Σ7: 59.6 ± 8.1			
		Outside backs		16.0 ± 0.2	178.8 ± 5.5	–	74.9 ± 7.6	Σ7: 65.4 ± 17.0			
Gabbett et al. [38]	Elite	All	28	16.0 ± 0.2	178.0 ± 5.9	–	77.5 ± 10.0	Σ7: 67.1 ± 14.8	Stadiometer	Electronic scales	Harpenden
Waldron et al. [46]	Academy	All	13	16.2 ± 0.3	–	94.6 ± 1.9	86.1 ± 6.0	–	Stadiometer	Electronic Scales	–
Baker et al. [105]	Academy	All	11	16.2 ± 1.2	184.0 ± 4.1	–	83.6 ± 7.4	–	NR	NR	–
Waldron et al. [52]	Academy	All	21	16.2 ± 0.3	179.3 ± 3.8	–	81.4 ± 8.7	–	NR	NR	–
Gabbett et al. [48]	Academy	All	15	16.5 ± 0.3	177.7 ± 6.2	–	78.5 ± 13.6	Σ4: 38.5 ± 19.1	Stadiometer	Electronic scales	Harpenden
Coutts et al. [64]	Academy	Al	21	16.6 ± 1.2	168.0 ± 6.4	–	74.7 ± 8.6	–	Stadiometer	Electronic scales	–
	Academy	All	21	16.8 ± 1.0	170.0 ± 5.4	–	77.9 ± 8.7	–			
Till et al. [61]	Academy	All	44	16.7 ± 0.2	178.7 ± 5.5	–	81.8 ± 9.3	Σ4: 37.0 ± 13.2	Stadiometer	Electronic Scales	Harpenden
Gabbett et al. [47]	Academy	All	21	16.9 ± 0.3	179.7 ± 1.3	–	80.1 ± 2.3	Σ7: 81.1 ± 5.7	Stadiometer	Electronic scales	Harpenden
Gabbet et al. [49]	Academy	All	36	16.9 ± 0.6	176.3 ± 6.5	–	74.3 ± 16.4	Σ7: 77.2 ± 34.3	Stadiometer	Electronic scales	Harpenden
Gabbett et al. [24]	Academy	All	25	16.9 ± 0.3	177.0 ± 6.1	–	74.0 ± 9.6	–	NR	NR	–
	Elite	All	18	16.8 ± 0.3	177.0 ± 8.1	–	80.1 ± 11.2	–			
Till et al. [96]	Academy	All	51	NR	177.0 ± 6.0	–	81.1 ± 9.4	Σ4: 36.5 ± 12.8	Stadiometer	Electronic Scales	Harpenden
		Forwards	28	NR	173.4 ± 4.1	–	75.4 ± 7.0	Σ4: 31.4 ± 6.2			
		Backs	23	NR	180.5 ± 5.5	–	85.7 ± 8.6	Σ4: 40.5 ± 15.1			
Till et al. [103]	Academy	All	37	NR	176.9 ± 5.5	–	79.9 ± 10.3	Σ4: 37.1 ± 14.3	Stadiometer	Electronic Scales	Harpenden
	Elite	All	13	NR	181.8 ± 3.1	–	84.5 ± 5.2	Σ4: 34.6 ± 6.9			
*17 years of age*											
Waldron et al. [46]	Academy	All	13	17.0 ± 0.3	–	94.7 ± 2.1	86.3 ± 9.4	–	–	Electronic Scales	
Waldron et al. [52]	Academy	All	15	17.0 ± 0.4	180.4 ± 4.2	–	86.3 ± 9.7	–	NR	NR	
Fernandes et al. [86]	Academy	All	23	17.1 ± 1.0	–	–	85.9 ± 10.4			Electronic Scales	
Baker et al. [105]	Academy	All	15	17.1 ± 0.6	182.0 ± 5.3	–	85.3 ± 9.6	–	NR	NR	
Dobbin et al. [42]	Academy	All	16	17.2 ± 0.7	179.9 ± 4.9	–	88.5 ± 10.1	Σ7: 88.1 ± 25.3	Stadiometer	Electronic Scales	Harpenden
Pearce et al. [72]	Academy	All	52	17.2 ± 0.5	179.9 ± 7.0	–	83.8 ± 11.2	–	Stadiometer	Electronic Scales	–
Till et al. [97]	Academy	All	61	17.3 ± 0.7	181.3 ± 6.0	–	83.9 ± 10	Σ4: 39.7 ± 14.7	Stadiometer	Electronic Scales	Harpenden
Dobbin et al. [62]	Academy	All	55	17.4 ± 0.5	181.3 ± 6.3	–	89.0 ± 11.6	–	Stadiometer	Electronic Scales	–
Dobbin et al. [104]	Academy	All	67	17.4 ± 1.0	179.7 ± 6.1	–	85.2 ± 10.7	–	NR	NR	–
Tredrea et al. [60]	Academy	All	41	17.4 ± 0.7	175.6 ± 5.1	91.2 ± 2.2	82.0 ± 11.9	Σ7: 92.5 ± 44.0	Stadiometer	Electronic scales	NR
	Elite	All	19	17.0 ± 0.7	175.7 ± 5.1	91.2 ± 2.4	86.7 ± 12.6	Σ7: 96.1 ± 35.2			
Dobbin et al. [65]	Academy	All	197	17.4 ± 1.0	179.4 ± 5.0	–	85.5 ± 7.4	–	Stadiometer	Electronic Scales	–
		Back Row		17.5 ± 0.8	182.1 ± 1.9	–	88.8 ± 4.8	–			
		Centre		17.2 ± 0.9	180.4 ± 4.9	–	85.0 ± 6.6	–			
		Forward		17.3 ± 0.5	182.5 ± 5.2	–	95.1 ± 14.7	–			
		Full back		17.4 ± 0.9	176.6 ± 5.3	–	80.1 ± 8.1	–			
		Half Back		17.3 ± 1.1	175.0 ± 4.3	–	76.8 ± 6.9	–			
		Hooker		17.2 ± 1.2	173.2 ± 3.9	–	77.2 ± 9.5	–			
		Loose Forward	17.5 ± 0.8	179.8 ± 3.8	–	88.4 ± 5.4	–				
		Prop		17.2 ± 0.9	183.2 ± 5.9	–	99.1 ± 8.6	–			
		Scrum Half		17.3 ± 1.4	172.7 ± 5.4	–	72.5 ± 5.6	–			
		Second Row		17.9 ± 1.4	184.9 ± 5.4	–	90.4 ± 5.6	–			
		Stand-Off^d^		18.0 ± 0.6	178.0 ± 4.5	–	88.5 ± 5.6	–			
		Winger		17.4 ± 1.1	183.8 ± 7.5	–	84.6 ± 7.5	–			
Dobbin et al. [66]	Academy	All	365	17.5 ± 2.0	180.7 ± 6.4	–	87.5 ± 11.7	–	Stadiometer	Electronic Scales	–
		Winger/Full Back	–	180.9 ± 6.5	–	82.2 ± 9.5	–	–	–	–	
		Centre		–	181.4 ± 5.4	–	85.3 ± 6.7	–	–	–	–
		Halves		–	176.4 ± 5.0	–	78.1 ± 6.8	–	–	–	–
		Hooker		–	173.8 ± 6.2	–	78.1 ± 8.7	–	–	–	–
		Prop		–	183.0 ± 6.1	–	99.7 ± 11.7	–	–	–	–
		Back Row		–	183.0 ± 4.9	–	90.9 ± 8.4	–	–	–	–
Gabbett et al. [22]^a^	Academy	Props	37	17.6 ± 2.4	183.9 ± 5.4	–	101.1 ± 11.0	Σ4: 72.0 ± 21.6	Stadiometer	Electronic scales	Harpenden
		Hookers	31	17.3 ± 1.1	171.9 ± 2.9	–	69.9 ± 5.7	Σ4: 35.9 ± 12.5	–	–	–
		Back Row	36	17.2 ± 1.8	176.8 ± 4.3	–	83.6 ± 9.6	Σ4: 39.5 ± 10.1	–	–	–
		Lock^d^	11	16.5 ± 1.5	176.7 ± 3.6	–	74.8 ± 7.0	Σ4: 33.7 ± 7.2			
		Halves^d^	27	16.7 ± 1.5	170.6 ± 6.5	–	69.1 ± 7.2	Σ4: 40.9 ± 7.2			
		Five Eighth^d^	11	16.7 ± 1.6	176.3 ± 2.8	–	72.0 ± 9.8	Σ4: 24.7 ± 1.6			
		Centre	27	17.0 ± 2.3	176.7 ± 2.9	–	79.6 ± 8.7	Σ4: 34.8 ± 9.9			
		Winger	39	17.7 ± 2.2	176.4 ± 4.2	–	72.9 ± 8.2	Σ4: 30.7 ± 7.4			
		Fullback	21	17.4 ± 2.0	177.1 ± 3.5	–	78.8 ± 11.9	Σ4: 36.2 ± 9.2			
Nicholson et al. [56]	Academy	All	20	17.6 ± 0.9	179.9 ± 6.6	–	91.2 ± 11.8	–	–	–	–
Till et al. [61]	Academy	All	34	17.7 ± 0.3	180.9 ± 5.2	–	87.3 ± 10.4	Σ4: 38.1 ± 13.0	Stadiometer	Electronic Scales	Harpenden
Darrel-Jones et al. [63]	Academy	All	14	17.7 ± 0.6	180.9 ± 6.4	–	85.9 ± 9.4	–	NR	NR	–
Gabbett et al. [1]^a^	Academy	Forwards	10	17.8 ± 0.8	–	–	89.4 ± 15.1	–	–	–	–
		Backs	12	17.3 ± 0.8	–	–	74.2 ± 10.2	–	–	–	–
Gabbett et al. [23]^a^	Academy	All	36	17.9 ± 0.4	177.0 ± 7.4	–	83.3 ± 32.7	Σ7: 93.9 ± 31.8	Stadiometer	Electronic scales	–
Till et al. [96]	Academy	All	61	NR	179.5 ± 5.8	–	85.3 ± 10.0	Σ4: 38.1 ± 12.1	Stadiometer	Electronic Scales	Harpenden
		Forwards	28	NR	176.7 ± 5.9	–	78.5 ± 7.6	Σ4: 32.2 ± 7.9	–	–	–
		Backs	33	NR	181.9 ± 4.6	–	90.9 ± 8.1	Σ4: 42.9 ± 12.8	–	–	–
Till et al. [59]	Academy	All	64	NR	–	–	84.1 ± 10.8	Σ4: 41.9 ± 17.6	–	Electronic Scales	Harpenden
Till et al. [103]	Academy	All	41	NR	179.0 ± 5.3	–	84.7 ± 10.7	Σ4: 39.0 ± 13.6	Stadiometer	Electronic Scales	Harpenden
	Elite	All	19	NR	181.8 ± 5.0	–	87.4 ± 8.9	Σ4: 36.1 ± 7.6			
*18 years of age*											
Till et al. [61]	Academy	All	16	18.7 ± 0.2	180.8 ± 4.8	–	88.4 ± 9.2	Σ4: 37.6 ± 10.3	Stadiometer	Electronic Scales	Harpenden
Kirkpatrick et al. [13]	Elite	Forwards	12	18.8 ± 1.1	180.1 ± 11.7	–	90.1 ± 11.7	–	NR	NR	–
		Backs	12	18.7 ± 0.8	176.8 ± 6.1	–	82.8 ± 6.3	–	–	–	–
Pearce et al. [72]	Academy	All	53	18.9 ± 0.6	179.2 ± 6.3	–	85.5 ± 11.1	–	Stadiometer	Electronic Scales	–
Till et al. [96]	Academy	All	50	NR	180.2 ± 2.7	–	88.0 ± 9.4	Σ4: 37 ± 10.6	Stadiometer	Electronic Scales	Harpenden
		Forwards	22	NR	179.3 ± 5.4	–	81.8 ± 8.0	Σ4: 30.5 ± 6.9			
		Backs	28	NR	182.7 ± 5.0	–	94.1 ± 7.7	Σ4: 43.5 ± 14.2			
Till et al. [103]	Academy	All	30	NR	180.5 ± 5.3	–	87.5 ± 9.9	Σ4: 38.4 ± 15.6	Stadiometer	Electronic Scales	Harpenden
	Elite	All	19	NR	182.3 ± 5.2	–	90.8 ± 9.7	Σ4: 36.9 ± 8.5			

*NR* not reported, – not tested, *All* players combined across all positions

^a^Reported 95% confidence intervals, which were converted to standard deviation according to the Cochrane Handbook for Systematic Reviews of Interventions Version 5.1.0

^b^Players classified as elite by definition and within ‘national’ squads as identified by authors

^c^Players classified as elite by definition and within ‘regional’ squads as identified by authors

^d^Exception to age grouping as a subsample of larger study sample within this age category

When examining the protocols used to assess anthropometric qualities, 33 studies (89%) reported standing height, of which 25 studies (76%) reported using a stadiometer (cm) and six studies (16%) did not report the equipment used. Thirty-seven studies (100%) reported body mass (kg), of which most studies (31 studies, 86%) reported using electronic scales, and six studies (16%) did not report the equipment used. Fourteen studies (39%) reported measuring body composition via skinfold thickness using Σ4 sites, of which all studies (14 studies, 100%) reported using Harpenden callipers.

### Linear Speed

Table 3 shows data for the included tests examining linear speed, measuring performance time (s) most frequently using the 10-m sprint test. A total of 37 studies examined linear speed in male, adolescent rugby league players aged between 12 and 19 years. Most studies examined 17-year-old players (18 studies, 55%) with the least frequently studied age group being 12-year-old players (one study, 3%). A total sample of 5789 players were included across all studies examining linear speed. Across all ages, studies most frequently assessed academy players (31 studies, 94%), followed by elite (11 studies, 33%), and amateur players (three studies, 9%). Nine studies (27%) reported data for players competing at multiple playing levels. Most studies (24 studies, 73%) did not report data according to playing position and grouped data for all players collectively.

**Table 3:** Linear sprint times (s) reported in male, adolescent rugby league players according to age group, playing level, and playing position

Study	Playing level	Position	Sample size (*n*)	Age (years)	Linear sprint distance					Methods		
					10 m (s)	20 m (s)	30 m (s)	40 m (s)	60 m (s)	Start distance from gate (m)	Number of trials (measure)	Equipment
*12 years of age*												
Gabbett et al. [1]^a^	Academy	Forwards	13	12.5 ± 0.5	2.60 ± 0.12	4.24 ± 0.17	–	7.50 ± 0.35	–	NR	2 (best)	LG
		Backs	14	12.3 ± 0.5	2.46 ± 0.14	4.04 ± 0.21	–	7.11 ± 0.42	–			
*13* years* of age*												
Gabbett et al. [48]	Academy	All	53	13.2 ± 0.6	2.14 ± 0.19	3.63 ± 0.23	–	6.60 ± 0.47	–	NR	3 (best)	LG
Gabbett et al. [1]^a^	Academy	Forwards	7	13.5 ± 0.5	2.44 ± 0.11	3.99 ± 0.22	–	7.00 ± 0.65	–	NR	2 (best)	LG
		Backs	10	13.7 ± 0.4	2.24 ± 0.13	3.70 ± 0.27	–	6.47 ± 0.55	–			
Till et al. [51]	Academy	All	194	13.6 ± 0.3	1.94 ± 0.12	3.36 ± 0.17	4.73 ± 0.26	–	8.86 ± 0.56	0.5	3 (best)	LG
Till et al. [8]	Amateur	All	249	13.6 ± 0.6	1.90 ± 0.14	3.29 ± 0.19	4.61 ± 0.27	–	8.60 ± 0.56	NR	3 (best)	LG
	Academy	All	261	13.6 ± 0.6	1.88 ± 0.13	3.25 ± 0.17	4.54 ± 0.23	–	8.43 ± 0.45			
	Elite	All	70	13.8 ± 0.7	1.85 ± 0.12	3.21 ± 0.16	4.51 ± 0.22	–	8.36 ± 0.49			
Till et al. [50]	Amateur	All	NR	13.6 ± 0.2	1.97 ± 0.09	3.41 ± 0.18	4.81 ± 0.26	–	9.17 ± 0.60	NR	NR	LG
	Academy	All	NR	13.7 ± 0.1	1.94 ± 0.06	3.32 ± 0.13	4.67 ± 0.20	–	8.75 ± 0.44			
	Elite	All	NR	13.4 ± 0.3	1.95 ± 0.09	3.34 ± 0.15	4.66 ± 0.23	–	8.69 ± 0.49			
Till et al. [102]	Academy	All	NR	13.6 ± 0.2	1.96 ± 0.08	3.36 ± 0.16	4.70 ± 0.23	–	8.76 ± 0.54	0.5	3 (best)	LG
		Outside backs	NR	13.7 ± 0.2	1.92 ± 0.09	3.28 ± 0.15	4.57 ± 0.23	–	8.44 ± 0.46			
		Pivots	NR	13.5 ± 0.3	1.95 ± 0.09	3.38 ± 0.16	4.74 ± 0.24	–	8.88 ± 0.56			
		Props	NR	13.6 ± 0.2	2.01 ± 0.08	3.47 ± 0.16	4.85 ± 0.24	–	9.10 ± 0.58			
		Backs	NR	13.7 ± 0.2	1.95 ± 0.06	3.34 ± 0.12	4.72 ± 0.15	–	8.82 ± 0.39			
Till et al. [39]	Amateur	All	50	13.6 ± 0.3	1.92 ± 0.13	3.37 ± 0.21	4.78 ± 0.28	–	9.07 ± 0.60	NR	3 (best)	LG
	Academy	All	32	13.6 ± 0.2	1.88 ± 0.09	3.29 ± 0.14	4.66 ± 0.20	–	8.74 ± 0.44			
	Elite	All	13	13.6 ± 0.3	1.87 ± 0.08	3.32 ± 0.14	4.70 ± 0.19	–	8.84 ± 0.36			
Till et al. [45]	Elite^b^	All	255	13.6 ± 0.3	1.95 ± 0.12	3.38 ± 0.20	4.75 ± 0.26	–	8.49 ± 0.55	NR	3 (best)	LG
	Elite^c^	All	130	13.7 ± 0.2	1.91 ± 0.12	3.29 ± 0.14	4.61 ± 0.22	–	–			
Till et al. [95]	Academy	All	NR	13.8 ± 0.1	1.95 ± 0.08	3.34 ± 0.14	4.67 ± 0.22	–	8.67 ± 0.50	0.5	3 (best)	LG
*14 years of age*												
Gabbett et al. [47]	Academy	All	14	14.1 ± 0.2	1.85 ± 0.02	3.20 ± 0.04	–	5.79 ± 0.08	–	NR	NR	LG
Till et al. [39]	Amateur	All	92	14.5 ± 0.3	1.93 ± 0.10	3.32 ± 0.15	4.63 ± 0.23	–	8.61 ± 0.48	NR	3 (best)	LG
	Academy	All	85	14.6 ± 0.3	1.90 ± 0.10	3.28 ± 0.15	4.58 ± 0.20	–	8.49 ± 0.44			
	Elite	All	18	14.5 ± 0.3	1.86 ± 0.09	3.24 ± 0.13	4.52 ± 0.17	–	8.39 ± 0.37			
Gabbett et al. [1]^a^	Academy	Forwards	11	14.5 ± 0.5	2.25 ± 0.07	3.72 ± 0.16	–	6.58 ± 0.39	–	NR	2 (best)	LG
		Backs	12	14.6 ± 0.5	2.21 ± 0.13	3.62 ± 0.14	–	6.26 ± 0.19	–			
Gabbett et al. [24]	Academy	All	23	14.5 ± 0.5	2.23 ± 0.12	–	–	6.41 ± 0.35	–	NR	2 (best)	LG
	Elite	All	36	14.3 ± 0.9	1.89 ± 0.12	–	–	5.65 ± 0.30	–			
Till et al. [51]	Academy	All	227	14.6 ± 0.3	1.89 ± 0.10	3.27 ± 0.16	4.56 ± 0.22	–	8.48 ± 0.47	0.5	3 (best)	LG
Till et al. [45]	Elite^b^	All	309	14.6 ± 0.3	1.91 ± 0.09	3.29 ± 0.25	4.58 ± 0.22	–	8.49 ± 0.46	NR	3 (best)	LG
	Elite^c^	All	86	14.6 ± 0.3	1.87 ± 0.10	3.21 ± 0.15	4.50 ± 0.21	–	8.32 ± 0.44			
Till et al. [50]	Amateur	All	NR	14.6 ± 0.2	1.95 ± 0.09	3.34 ± 0.15	4.67 ± 0.23	–	8.62 ± 0.51	NR	NR	LG
	Academy	All	NR	14.7 ± 0.1	1.91 ± 0.07	3.23 ± 0.11	4.50 ± 0.17	–	8.28 ± 0.32			
	Elite	All	NR	14.4 ± 0.3	1.88 ± 0.10	3.22 ± 0.15	4.49 ± 0.21	–	8.33 ± 0.41			
Till et al. [102]	Academy	All	NR	14.6 ± 0.2	1.91 ± 0.08	3.26 ± 0.14	4.53 ± 0.20	–	8.39 ± 0.41	0.5	3 (best)	LG
		Outside backs	NR	14.7 ± 0.2	1.87 ± 0.07	3.18 ± 0.11	4.41 ± 0.15	–	8.14 ± 0.30			
		Pivots	NR	14.5 ± 0.3	1.92 ± 0.10	3.28 ± 0.17	4.56 ± 0.25	–	8.49 ± 0.52			
		Props	NR	14.6 ± 0.2	1.95 ± 0.07	3.34 ± 0.11	4.66 ± 0.17	–	8.65 ± 0.35			
		Backs	NR	14.7 ± 0.2	1.90 ± 0.07	3.26 ± 0.12	4.54 ± 0.17	–	8.39 ± 0.34			
Till et al. [95]	Academy	All	NR	14.8 ± 0.1	1.91 ± 0.08	3.25 ± 0.13	4.51 ± 0.19	–	8.34 ± 0.38	0.5	3 (best)	LG
Till et al. [59]	Academy	All	31	NR^g^	1.94 ± 0.10	3.37 ± 0.17	–	–	–	0.5	3 (best)	LG
*15 years of age*												
Dobbin et al. [66]	Academy	All	235	15.1 ± 0.8	1.83 ± 0.11	3.16 ± 0.16	–	–	–	0.3	2 (best)	LG
		Wingers	-	15.1 ± 0.8	1.82 ± 0.09	3.12 ± 0.14	–	–	–			
		Centres	-	15.1 ± 0.8	1.81 ± 0.12	3.13 ± 0.15	–	–	–			
		Halves	-	15.1 ± 0.9	1.83 ± 0.13	3.19 ± 0.18	–	–	–			
		Hookers	-	15.1 ± 0.1	1.85 ± 0.10	3.21 ± 0.17	–	–	–			
		Hit-up forwards	-	15.1 ± 0.1	1.87 ± 0.11	3.22 ± 0.15	–	–	–			
		Backs	-	15.1 ± 0.1	1.82 ± 0.11	3.15 ± 0.16	–	–	–			
Gabbett et al. [48]	Academy	All	20	15.1 ± 0.6	1.97 ± 0.13	3.34 ± 0.25	–	5.97 ± 0.54	–	NR	3 (best)	LG
Waldron et al. [46]	Academy	All	13	15.1 ± 0.3	–	3.50 ± 0.10	–	–	–	0.3	2 (best)	LG
Gabbett et al. [1]^a^	Academy	Forwards	12	15.4 ± 0.9	2.22 ± 0.11	3.61 ± 0.16	–	6.17 ± 0.27	–	NR	2 (best)	LG
		Backs	9	15.4 ± 0.5	2.17 ± 0.09	3.55 ± 0.12	–	6.00 ± 0.17	–			
Tredrea et al. [60]	Academy	All	49	15.5 ± 0.6	1.93 ± 0.11	–	–	5.75 ± 0.29	–	0.5	2 (best)	LG
	Elite	All	51	15.5 ± 0.5	1.85 ± 0.12	–	–	5.56 ± 0.26	–			
Till et al. [51]	Academy	All	208, 178^f^	15.5 ± 0.3	1.86 ± 0.16	3.10 ± 0.18	4.45 ± 0.23	–	8.24 ± 0.37	0.5	3 (best)	LG
Till et al. [50]	Amateur	All	NR	15.6 ± 0.2	1.89 ± 0.08	3.22 ± 0.12	4.50 ± 0.17	–	8.27 ± 0.36	NR	NR	LG
	Academy	All	NR	15.7 ± 0.1	1.89 ± 0.07	3.22 ± 0.15	4.44 ± 0.18	–	8.19 ± 0.39			
	Elite	All	NR	15.4 ± 0.3	1.86 ± 0.10	3.18 ± 0.14	4.38 ± 0.22	–	8.09 ± 0.42			
Gabbett et al. [24]	Academy	All	28	15.6 ± 0.6	2.15 ± 0.17	–	–	6.01 ± 0.34	–	NR	2 (best)	LG
	Elite	All	21	15.8 ± 0.3	1.81 ± 0.07	–	–	5.42 ± 0.25	–			
Till et al. [102]	Academy	All	NR	15.6 ± 0.2	1.87 ± 0.08	3.18 ± 0.12	4.41 ± 0.19	–	8.10 ± 0.41	0.5	3 (best)	LG
		Outside backs	NR	15.7 ± 0.2	1.83 ± 0.07	3.10 ± 0.11	4.28 ± 0.16	–	7.80 ± 0.35			
		Pivots	NR	15.5 ± 0.3	1.87 ± 0.07	3.19 ± 0.10	4.45 ± 0.17	–	8.19 ± 0.35			
		Props	NR	15.6 ± 0.2	1.91 ± 0.07	3.25 ± 0.11	4.48 ± 0.17	–	8.30 ± 0.29			
		Backs	NR	15.7 ± 0.2	1.88 ± 0.08	3.21 ± 0.11	4.49 ± 0.17	–	8.27 ± 0.39			
Till et al. [39]	Amateur	All	107	15.6 ± 0.3	1.87 ± 0.16	3.21 ± 0.19	4.47 ± 0.24	–	8.28 ± 0.42	NR	3 (best)	LG
	Academy	All	144	15.6 ± 0.3	1.87 ± 0.15	3.20 ± 0.18	4.46 ± 0.24	–	8.23 ± 0.41			
	Elite	All	39	15.5 ± 0.3	1.82 ± 0.14	3.14 ± 0.15	4.38 ± 0.17	–	8.10 ± 0.30			
Till et al. [45]	Elite^b^	All	306	15.6 ± 0.3	1.86 ± 0.14	3.19 ± 0.17	4.43 ± 0.22	–	8.17 ± 0.41	NR	3 (best)	LG
	Elite^c^	All	86	15.6 ± 0.3	1.85 ± 0.14	3.17 ± 0.17	4.40 ± 0.23	–	8.10 ± 0.43			
Till et al. [61]	Academy	All	35	15.7 ± 0.2	1.81 ± 0.07	3.12 ± 0.11	–	–	–	0.5	3 (best)	LG
Till et al. [95]	Academy	All	NR	15.8 ± 0.1	1.88 ± 0.08	3.19 ± 0.12	4.40 ± 0.17	–	8.07 ± 0.37	0.5	3 (best)	LG
Gabbett et al. [14]	Academy	All	36	15.9 ± 0.6	1.94 ± 0.11	3.28 ± 0.18	–	5.83 ± 0.35	–	NR	3 (best)	LG
		Hit-up forwards	15.8 ± 0.7	2.06 ± 0.09	3.50 ± 0.16	–	6.28 ± 0.31	–				
		Adjustables		15.7 ± 0.4	1.91 ± 0.06	3.25 ± 0.08	–	5.77 ± 0.19	–			
		Outside backs^e^		16.1 ± 0.7	1.88 ± 0.07	3.18 ± 0.09	–	5.62 ± 0.15	–			
Gabbett et al. [38]	Academy	All	13	15.9 ± 0.6	1.94 ± 0.13					0	3 (best)	LG
Till et al. [96]	Academy	All	67	NR^h^	1.82 ± 0.07	3.13 ± 0.11	–	–	–	0.5	3 (best)	LG
		Forwards	37	NR^h^	1.85 ± 0.06	3.18 ± 0.09	–	–	–			
		Backs	30	NR^h^	1.78 ± 0.06	3.07 ± 0.11	–	–	–			
*16 years of age*												
Gabbett et al. [14]	Academy	All^d^	36	15.9 ± 0.6	1.94 ± 0.11	3.28 ± 0.18	–	5.83 ± 0.35	–	NR	3 (best)	LG
		Hit-up forwards		15.8 ± 0.7	2.06 ± 0.09	3.50 ± 0.16	–	6.28 ± 0.31	–			
		Adjustables		15.7 ± 0.4	1.91 ± 0.06	3.25 ± 0.08	–	5.77 ± 0.19	–			
		Outside backs^e^		16.1 ± 0.7	1.88 ± 0.07	3.18 ± 0.09	–	5.62 ± 0.15	–			
	Elite	All^d^	28	16.0 ± 0.2	1.81 ± 0.08	3.11 ± 0.12	–	5.56 ± 0.22	–	NR	3 (best)	LG
		Hit-up forwards		15.9 ± 0.4	1.85 ± 0.06	3.18 ± 0.09	–	5.69 ± 0.17	–			
		Adjustables^e^		16.0 ± 0.2	1.81 ± 0.06	3.12 ± 0.11	–	5.58 ± 0.20	–			
		Outside backs^e^		16.0 ± 0.2	1.76 ± 0.09	3.03 ± 0.12	–	5.40 ± 0.22	–			
Gabbett et al. [38]	Academy	All	28	16.0 ± 0.2	1.81 ± 0.08		–	–	–	0	3 (best)	LG
Waldron et al. [46]	Academy	All	13	16.2 ± 0.3	–	3.4 ± 0.2	–	–	–	0.3	2 (best)	LG
Gabbett et al. [48]	Academy	All	15	16.5 ± 0.3	1.94 ± 0.09	3.26 ± 0.18	–	5.80 ± 0.39	–	NR	3 (best)	LG
Till et al. [61]	Academy	All	44	16.7 ± 0.2	1.80 ± 0.06	3.11 ± 0.09	–	–	–	0.5	3 (best)	LG
Coutts et al. [64]	Academy	All	21	16.6 ± 1.2	2.14 ± 0.09	3.47 ± 0.11	–	–	–	NR	2 (best)	LG
	Academy	All	21	16.8 ± 1.0	2.16 ± 0.08	3.46 ± 0.08	–	–	–			
Gabbet et al. [49]	Academy	All	36	16.9 ± 0.6	1.80 ± 0.07	3.08 ± 0.09	–	5.48 ± 0.21	–	NR	2 (best)	LG
Gabbett et al. [47]	Academy	All	21	16.9 ± 0.3	1.83 ± 0.02	3.14 ± 0.03	–	5.64 ± 0.06	–	NR	NR	LG
Gabbett et al.[24]	Academy	All	25	16.9 ± 0.3	2.08 ± 0.19	–	–	5.92 ± 0.34		NR	2 (best)	LG
	Elite	All	18	16.8 ± 0.3	1.83 ± 0.11	–	–	5.46 ± 0.29	–			
Till et al. [96]	Academy	All	45	NR^i^	1.81 ± 0.06	3.12 ± 0.10	–	–	–	0.5	3 (best)	LG
		Forwards	25	NR^i^	1.83 ± 0.07	3.16 ± 0.11	–	–	–			
		Backs	20	NR^i^	1.78 ± 0.04	3.07 ± 0.07	–	–	–			
Till et al. [103]	Academy	All	37	NR	1.81 ± 0.06	3.12 ± 0.09	–	–	–	0.5	3 (best)	LG
	Elite	All	13	NR	1.80 ± 0.05	3.10 ± 0.09	–	–	–			
Till et al. [59]	Academy	All	75	NR^i^	1.83 ± 0.07	3.16 ± 0.12	–	–	–	0.5	3 (best)	LG
*17 years of age*												
Waldron et al. [46]	Academy	All	13	17.0 ± 0.3	–	3.3 ± 0.1	–	–	–	0.3	2 (best)	LG
Dobbin et al. [62]	Academy	All	50	17.1 ± 1.1	1.90 ± 0.11	3.23 ± 0.20	–	–	–	0.3	2 (best)	LG
Dobbin et al. [42]	Academy	All	16	17.2 ± 0.7	1.79 ± 0.08	3.06 ± 0.12	–	–	–	0.3	2 (best)	LG
Pearce et al. [72]	Academy	All	52	17.2 ± 0.5	–	–	4.31 ± 0.16	–	–	NR	3 (best)	LG
Till et al. [97]	Academy	All	16	17.3 ± 0.7	1.81 ± 0.1	3.11 ± 0.1	–	–	–	0.5	3 (best)	LG
Dobbin et al. [65]	Academy	All	197	17.4 ± 1.0	1.84 ± 0.11	3.10 ± 0.15	–	–	–	0.3	2 (best)	LG
		Back Row		17.5 ± 0.8	1.80 ± 0.08	3.05 ± 0.13	–	–	–			
		Centre		17.2 ± 0.9	1.81 ± 0.08	3.06 ± 0.11	–	–	–			
		Forward		17.3 ± 0.5	1.86 ± 0.16	3.12 ± 0.16	–	–	–			
		Full back		17.4 ± 0.9	1.81 ± 0.12	3.05 ± 0.11	–	–	–			
		Half Back		17.3 ± 1.1	1.83 ± 0.10	3.08 ± 0.22	–	–	–			
		Hooker		17.2 ± 1.2	1.83 ± 0.11	3.06 ± 0.20	–	–	–			
		Loose Forward		17.5 ± 0.8	1.87 ± 0.10	3.15 ± 0.14	–	–	–			
		Prop		17.2 ± 0.9	1.89 ± 0.13	3.23 ± 0.15	–	–	–			
		Scrum Half		17.3 ± 1.4	1.82 ± 0.12	3.05 ± 0.13	–	–	–			
		Second Row		17.9 ± 1.4	1.83 ± 0.12	3.11 ± 0.13	–	–	–			
		Stand-Off		18.0 ± 0.6	1.91 ± 0.14	3.22 ± 0.15	–	–	–			
		Winger		17.4 ± 1.1	1.76 ± 0.07	3.00 ± 0.13	–	–	–			
Dobbin et al. [104]	Academy	All	67	17.4 ± 1.0	1.86 ± 0.07	3.15 ± 0.10	4.36 ± 0.14	–	–	0.3	2 (worse)	LG
Tredrea et al. [60]	Academy	All	41	17.4 ± 0.7	1.86 ± 0.08	–	–	5.57 ± 0.23	–	0.5	2 (best)	LG
	Elite	All	19	17.0 ± 0.7	1.76 ± 0.07	–	–	5.44 ± 0.20	–			
Dobbin et al. [66]	Elite	All^d^	365	17.5 ± 2.0	1.84 ± 0.11	3.15 ± 0.16	–	–	–	0.3	2 (best)	LG
		Wingers		17.5 ± 2.0	1.80 ± 0.09	3.08 ± 0.15	–	–	–			
		Centres		17.5 ± 2.0	1.81 ± 0.09	3.10 ± 0.13	–	–	–			
		Halves		17.5 ± 2.1	1.83 ± 0.09	3.12 ± 0.14	–	–	–			
		Hookers		17.5 ± 2.2	1.83 ± 0.09	3.11 ± 0.16	–	–	–			
		Hit-up forwards		17.5 ± 2.3	1.91 ± 0.10	3.28 ± 0.15	–	–	–			
		Backs		17.5 ± 2.4	1.85 ± 0.12	3.16 ± 0.15	–	–	–			
Gabbett et al. [22]^a^	Academy	Props	37	17.6 ± 2.4	2.14 ± 0.19	3.62 ± 0.21	–	6.18 ± 0.42	–	NR	2 (best)	LG
		Hookers	31	17.3 ± 1.1	2.04 ± 0.22	3.38 ± 0.29	–	5.89 ± 0.38	–			
		Back rowers	36	17.2 ± 1.8	2.07 ± 0.27	3.47 ± 0.31	–	5.89 ± 0.49	–			
		Locks^e^	11	16.5 ± 1.5	2.07 ± 0.18	3.49 ± 0.22	–	5.93 ± 0.36	–			
		Halves^e^	27	16.7 ± 1.5	1.95 ± 0.21	3.52 ± 0.10	–	5.62 ± 0.38	–			
		Five eighth^e^	11	16.7 ± 1.6	1.93 ± 0.16	3.27 ± 0.06	–	5.71 ± 0.16	–			
		Centres	27	17.0 ± 2.3	2.02 ± 0.23	3.34 ± 0.23	–	5.71 ± 0.33	–			
		Wingers	39	17.7 ± 2.2	2.18 ± 0.26	3.49 ± 0.28	–	5.94 ± 0.51	–			
		Fullbacks	21	17.4 ± 2.0	2.16 ± 0.13	3.39 ± 0.22	–	5.84 ± 0.21	–			
Nicholson et al. [56]	Academy	All	20	17.6 ± 0.9	2.15 ± 0.12	3.46 ± 0.18	4.68 ± 0.24	5.87 ± 0.30	–	NR	2	RG
		Forwards			2.17 ± 0.13	3.50 ± 0.19	4.73 ± 0.24	5.96 ± 0.29	–			
		Backs			2.12 ± 0.11	3.38 ± 0.16	4.57 ± 0.21	5.71 ± 0.27	–			
Till et al. [61]	Academy	All	34	17.7 ± 0.3	1.80 ± 0.06	3.09 ± 0.11	–	–	–	0.5	3 (best)	LG
Darrel-Jones et al. [63]	Academy	All	14	17.7 ± 0.6	1.79 ± 0.04	3.10 ± 0.06	4.30 ± 0.10	5.57 ± 0.11	–	0.5	3 (best)	LG
Gabbett et al. [1]^a^	Academy	Forwards	10	17.8 ± 0.8	2.19 ± 0.13	3.57 ± 0.15	–	6.20 ± 0.24	–	NR	2 (best)	LG
		Backs	12	17.3 ± 0.8	2.19 ± 0.16	3.53 ± 0.19	–	6.01 ± 0.25	–			
Gabbett et al. [23]^a^	Academy	All	36	17.9 ± 0.4	1.82 ± 0.25	3.12 ± 0.43	–	5.56 ± 0.87	–	NR	2 (best)	LG
Till et al. [59]	Academy	All	64	NR^j^	1.82 ± 0.06	3.14 ± 0.10	–	–	–	0.5	3 (best)	LG
Till et al. [96]	Academy	All	49	NR^j^	1.80 ± 0.06	3.09 ± 0.10	–	–	–	0.5	3 (best)	LG
		Forwards	27	NR^j^	1.81 ± 0.06	3.11 ± 0.11	–	–	–			
		Backs	22	NR^j^	1.78 ± 0.05	3.06 ± 0.09	–	–	–			
Till et al. [103]	Academy	All	41	NR^j^	1.80 ± 0.06	3.11 ± 0.10	–	–	–	0.5	3 (best)	LG
	Elite	All	19	NR^j^	1.79 ± 0.06	3.06 ± 0.10	–	–	–			
*18 years of age*												
Till et al. [61]	Academy	All	16	18.7 ± 0.2	1.81 ± 0.08	3.09 ± 0.13	–	–	–	0.5	3 (best)	LG
Kirkpatrick et al. [13]	Elite	Forwards	12	18.8 ± 1.1	2.06 ± 0.10	3.39 ± 0.17	–	5.80 ± 0.26	–	0.5	3 (best)	LG
		Backs	12	18.7 ± 0.8	1.99 ± 0.6	3.26 ± 0.70	–	5.55 ± 0.13	–			
Pearce et al. [72]	Academy	All	53	18.9 ± 0.6	–	–	4.21 ± 0.20	–	–	NR	3 (best)	LG
Till et al. [96]	Academy	All	39	NR^k^	1.82 ± 0.07	3.11 ± 0.12	–	–	–	0.5	3 (best)	LG
		Forwards	23	NR^k^	1.83 ± 0.08	3.14 ± 0.12	–	–	–			
		Backs	16	NR^k^	1.79 ± 0.07	3.04 ± 0.08	–	–	–			
Till et al. [103]	Academy	All	30	NR^k^	1.80 ± 0.05	3.10 ± 0.09	–	–	–	0.5	3 (best)	LG
	Elite	All	19	NR^k^	1.80 ± 0.09	3.09 ± 0.14	–	–	–			

*NR* not reported, – not tested, *All* players combined across all positions, *LG* electronic light gates used for measuring sprint time, *RG* radar gun used for measuring sprint time

^a^Reported 95% confidence intervals, which were converted to standard deviation according to the Cochrane Handbook for Systematic Reviews of Interventions Version 5.1.0

^b^Players classified as elite by definition used in this review but as ‘regional’ by the authors

^c^Players classified as elite by definition used in this review but as ‘national’ by the authors

^d^Total sample size (all positions combined) was reported rather than independently for each position

^e^Sample age reported across multiple years, and across various playing positions (data allocated according to weighted mean age (15.8 years of age))

^f^Sample size for each sprint distance varied so data are reported separately for 10-m (*n* = 208), 20-m (*n* = 208), 30-m (*n* = 178), 40-m (*n* = 178) and 60-m (*n* = 178) sprint trials in order

^g^Age provided categorically as under 14 years instead of being reported as mean ± standard deviation

^h^Age provided categorically as under 15 years instead of being reported as mean ± standard deviation

^i^Age provided categorically as under 16 years instead of being reported as mean ± standard deviation

^j^Age provided categorically as under 17 years instead of being reported as mean ± standard deviation

^k^Age provided categorically as under 18 years instead of being reported as mean ± standard deviation

When examining the protocols used to assess linear speed, 18 studies (55%) reported the starting distance of players behind the starting gate (0.3 m, *n* = 6, 33%; 0.5 m, *n* = 11, 61%; 0 m, *n* = 1, 6%) and 15 studies (45%) did not report where players started. Thirty-two studies (97%) reported linear sprint time in seconds (s) using electronic light gates, while one study (3%) reported sprint time in seconds (s) using a radar gun. Seventeen studies (52%) reported the best sprint time across three trials and 13 studies (39%) reported the best sprint time across two trials. One study (3%) reported that two trials were used in their analysis but did not indicate whether the best or average time was reported, and two studies (6%) did not report the number of trials completed, or how performance time was determined.

### Change of Direction Speed

Table 4 shows data for the included tests examining COD speed, measuring performance time (s) most frequently using the 505 Agility Test. A total of 16 studies examined COD speed in male, adolescent rugby league players aged between 13 and 18 years. Most studies examined 15-year-old players (10 studies, 63%), with the least frequently studied age group being 18-year-old players (one study, 6%). No included studies examined 12-year-old players. A total sample of 3765 players were included across all studies examining COD speed. Across all ages, studies most frequently assessed academy players (11 studies, 69%), followed by elite (five studies, 31%), and amateur players (one study, 6%). Four studies (25%) reported data for players competing at multiple playing levels. Most studies (13 studies, 81%) did not report data according to playing position and grouped data for all players collectively.

**Table 4:** Change-of-direction speed test times (s) reported in male, adolescent rugby league players according to age group, playing level, and playing position

Study	Playing level	Position	Sample size (*n*)	Age (years)	505 Agility Test time right foot (s)	505 Agility Test time left foot (s)	L-run Test time (s)	Methods	
								Trials (measure)	Equipment
*13 years of age*									
Gabbett et al. [48]	Academy	All	53	13.2 ± 0.6	2.67 ± 0.20^e^			3 (best)	LG
Till et al. [50]	Amateur	All	NR	13.6 ± 0.2	2.61 ± 0.18	2.60 ± 0.13	–	3 (NR)	NR
	Academy	All	NR	13.7 ± 0.1	2.51 ± 0.16	2.50 ± 0.15	–		
	Elite	All	NR	13.4 ± 0.3	2.57 ± 0.13	2.56 ± 0.12	–		
Till et al. [102]	Academy	All	NR	13.6 ± 0.2	2.57 ± 0.15	2.56 ± 0.13		NR (NR)	LG
		Outside backs	NR	13.7 ± 0.2	2.55 ± 0.15	2.55 ± 0.12			
		Pivots	NR	13.5 ± 0.3	2.56 ± 0.16	2.57 ± 0.17			
		Props	NR	13.6 ± 0.2	2.62 ± 0.12	2.61 ± 0.12			
		Backs	NR	13.7 ± 0.2	2.55 ± 0.12	2.55 ± 0.12			
Till et al. [39]	Amateur	All	50	13.6 ± 0.3	2.58 ± 0.16	2.58 ± 0.15		3 (best)	LG
	Academy	All	32	13.6 ± 0.2	2.49 ± 0.15	2.48 ± 0.13			
	Elite	All	13	13.6 ± 0.3	2.56 ± 0.15	2.50 ± 0.15			
Till et al. [51]	Academy	All	207	13.6 ± 0.3	2.57 ± 0.15	2.57 ± 0.15	–	3 (best)	LG
Till et al. [45]	Elite^b^	All	255	13.6 ± 0.3	2.61 ± 0.14	2.59 ± 0.14	–	3 (NR)	LG
	Elite^c^	All	130	13.7 ± 0.3	2.57 ± 0.14	2.55 ± 0.14	–		
Till et al. [8]	Amateur	All	249	13.6 ± 0.6	2.54 ± 0.15	2.52 ± 0.16	–	3 (best)	LG
	Academy	All	261	13.6 ± 0.6	2.48 ± 0.14	2.47 ± 0.13	–		
	Elite	All	70	13.8 ± 0.7	2.46 ± 0.12	2.42 ± 0.12	–		
Till et al. [95]	Academy	All	NR	13.8 ± 0.1	2.57 ± 0.13	2.55 ± 0.11	–	3 (best)	LG
*14 years of age*									
Gabbett et al. [47]	Academy	All	14	14.1 ± 0.3	2.42 ± 0.03^e^		–	NR (NR)	LG
Gabbett et al. [50]	Amateur	All	NR	14.6 ± 0.2	2.53 ± 0.16	2.52 ± 0.13			
	Academy	All	NR	14.7 ± 0.1	2.46 ± 0.12	2.48 ± 0.12			
	Elite	All	NR	14.4 ± 0.3	2.48 ± 0.11	2.47 ± 0.10			
Gabbett et al. [24]	Academy	All	23	14.5 ± 0.5	2.89 ± 0.10^e^	–	–	2 (best)	LG
	Elite	All	36	14.3 ± 0.9	2.45 ± 0.12^e^	–	–		
Till et al. [51]	Academy	All	226	14.6 ± 0.3	2.50 ± 0.16	2.48 ± 0.14	–	3 (best)	LG
Till et al. [45]	Elite^b^	All	309	14.6 ± 0.3	2.51 ± 0.15	2.49 ± 0.14	–	3 (NR)	LG
	Elite^c^	All	86	14.6 ± 0.3	2.46 ± 0.11	2.44 ± 0.12	–		
Till et al. [102]	Academy	All	NR	14.6 ± 0.2	2.47 ± 0.10	2.44 ± 0.13		NR (NR)	LG
		Outside backs	NR	14.7 ± 0.2	2.44 ± 0.11	2.43 ± 0.10			
		Pivots	NR	14.5 ± 0.3	2.47 ± 0.11	2.47 ± 0.07			
		Props	NR	14.6 ± 0.2	2.60 ± 0.08	2.59 ± 0.08			
		Backs	NR	14.7 ± 0.2	2.47 ± 0.14	2.46 ± 0.10			
Till et al. [39]	Amateur	All	92	14.6 ± 0.3	2.54 ± 0.17	2.52 ± 0.16		3 (best)	LG
	Academy	All	85	14.6 ± 0.3	2.49 ± 0.15	2.46 ± 0.13			
	Elite	All	18	14.5 ± 0.3	2.42 ± 0.09	2.38 ± 0.09			
Till et al. [95]	Academy	All	NR	14.8 ± 0.1	2.46 ± 0.12	2.44 ± 0.12	–	3 (best)	LG
*15 years of age*									
Gabbett et al. [48]	Academy	All	20	15.1 ± 0.6	2.43 ± 0.25^e^			NR (NR)	LG
Till et al. [51]	Academy	All	204	15.5 ± 0.3	2.51 ± 0.14	2.47 ± 0.16	–	3 (best)	LG
Till et al. [102]	Academy	All	NR	15.6 ± 0.2	2.47 ± 0.14	2.44 ± 0.13		NR (NR)	LG
		Outside backs	NR	15.7 ± 0.2	2.41 ± 0.11	2.40 ± 0.11			
		Pivots	NR	15.5 ± 0.3	2.46 ± 0.14	2.45 ± 0.12			
		Props	NR	15.6 ± 0.2	2.55 ± 0.17	2.49 ± 0.17			
		Backs	NR	15.7 ± 0.2	2.47 ± 0.14	2.42 ± 0.13			
Till et al. [39]	Amateur	All	107	15.6 ± 0.3	2.52 ± 0.13	2.49 ± 0.16		3 (best)	LG
	Academy	All	144	15.6 ± 0.3	2.50 ± 0.14	2.47 ± 0.15			
	Elite	All	39	15.5 ± 0.3	2.45 ± 0.11	2.42 ± 0.13			
Till et al. [45]	Elite^b^	All	306	15.6 ± 0.3	2.48 ± 0.13	2.46 ± 0.14	–	3 (NR)	LG
	Elite^c^	All	86	15.6 ± 0.3	2.47 ± 0.14	2.45 ± 0.16	–		
Gabbett et al. [24]	Academy	All	28	15.6 ± 0.6	2.85 ± 0.13^e^	–	–	2 (best)	LG
	Elite	All	21	15.8 ± 0.3	2.40 ± 0.11^e^	–	–		
Till et al. [50]	Amateur	All	NR	15.6 ± 0.2	2.48 ± 0.23	2.52 ± 0.19	–	3 (NR)	NR
	Academy	All	NR	15.7 ± 0.1	2.46 ± 0.12	2.53 ± 0.14	–		
	Elite	All	NR	15.4 ± 0.3	2.41 ± 0.11	2.43 ± 0.09	–		
Gabbett et al. [14]	Academy	All	36	15.6 ± 0.6	2.38 ± 0.16^e^		–	3 (best)	LG
	Academy	Hit-up forwards	15.8 ± 0.7	2.57 ± 0.08^e^		–			
	Academy	Adjustables		15.7 ± 0.4	2.27 ± 0.08^e^		–		
	Academy	Outside backs^d^		16.1 ± 0.7	2.33 ± 0.12^e^		–		
	Elite	All^d^	28	16.0 ± 0.2	2.30 ± 0.13^e^		–		
	Elite	Hit-up forwards	15.9 ± 0.4	2.34 ± 0.15^e^		–			
	Elite	Adjustables^d^		16.0 ± 0.2	2.30 ± 0.12^e^		–		
	Elite	Outside backs^d^		16.0 ± 0.2	2.27 ± 0.12^e^		–		
Till et al. [95]^e^	Academy	All	NR	15.8 ± 0.1	2.44 ± 0.16	2.43 ± 0.14	–	3 (best)	LG
Gabbett et al. [38]	Academy	All	13	15.9 ± 0.6	2.37 ± 0.13^e^			3 (best)	LG
*16 years of age*									
Gabbett et al. [38]	Academy	All	28	16.0 ± 0.2	2.30 ± 0.13^e^			3 (best)	LG
Gabbett et al. [48]	Academy	All	15	16.5 ± 0.3	2.37 ± 0.17^e^			NR (NR)	LG
Gabbett et al. [47]	Academy	All	21	16.9 ± 0.3	2.42 ± 0.02^e^		–	NR (NR)	LG
Gabbett et al. [49]	Academy	All	36	16.9 ± 0.6	–		5.81 ± 0.3	2 (best)	LG
Gabbett et al. [24]	Academy	All	25	16.9 ± 0.3	2.68 ± 0.20^e^	–	–	2 (best)	LG
	Elite	All	18	16.8 ± 0.3	2.36 ± 0.17^e^	–	–	2 (best)	LG
*17 years of age*									
Pearce et al. [72]	Academy	All	52	17.2 ± 0.5	–		8.60 ± 0.40	3 (best)	LG
Gabbett et al. [22]	Academy	Props	37	17.6 ± 2.4	–		6.37 ± 0.46	2 (best)	LG
		Hookers	31	17.3 ± 1.1	–		5.86 ± 0.68		
		Second rowers	36	17.2 ± 1.8	–		6.10 ± 0.58		
		Locks^d^	11	16.5 ± 1.5	–		5.64 ± 0.44		
		Halfbacks^d^	27	16.7 ± 1.5	–		6.01 ± 0.57		
		Five eighths^d^	11	16.7 ± 1.6	–		5.71 ± 0.55		
		Centres	27	17.0 ± 2.3	–		5.89 ± 0.52		
		Wingers	39	17.7 ± 2.2	–		5.98 ± 0.42		
		Fullbacks	21	17.4 ± 2.0	–		5.90 ± 0.40		
Gabbett et al. [23]^a^	Academy	All	36	17.9 ± 0.4	–		5.93 ± 0.64	2 (best)	LG
*18 years of age*									
Pearce et al. [72]	Academy	All	53	18.9 ± 0.6	–		8.7 ± 0.4	3 (best)	LG

*NR* not reported, – not tested, *All* players combined across all positions, *LG* electronic light gates used for measuring performance time

^a^Reported 95% confidence intervals, which were converted to standard deviation according to the Cochrane Handbook for Systematic Reviews of Interventions Version 5.1.0

^b^Players classified as elite by definition used in this review but as ‘regional’ by the authors

^c^Players classified as elite by definition used in this review but as ‘national’ by the authors

^d^Sample age reported across multiple years, and across various playing positions (data allocated according to weighted mean age)

^e^Foot selection not identified

When examining the protocols used to assess COD speed, eight studies (50%) reported the best performance time across three trials, and two studies (13%) used three trials but did not report how performance time was determined (i.e., mean or best). Four studies (25%) reported the best performance time across two trials, and two studies (13%) did not report the number of trials completed or how performance was determined. Fifteen studies (94%) reported COD performance time in seconds (s) using electronic light gates, and one study (6%) did not report the equipment used to measure performance time.

### Aerobic Capacity

Table 5 shows data for the included tests examining proxy measures of aerobic capacity, predicting maximum rate of oxygen consumption ($\dot{\mathrm{V}}$O_2max_ in mL kg^−1^ min^−1^) most frequently using the MSFT. A total of 27 studies examined estimated aerobic capacity in male, adolescent rugby league players aged between 12 and 19 years; one study [24] (4%) did not report predicted $\dot{\mathrm{V}}$O_2max_, and instead reported MSFT level obtained for 14-year-old players (academy = 8.0 ± 1.4; elite = 10.6 ± 1.5), 15-year-old players (academy = 9.4 ± 1.6; elite = 11.3 ± 1.5) and 16-year-old players (academy = 9.5 ± 1.8; elite = 12.3 ± 1.1). Most studies examined 17-year-old players (16 studies, 59%) with the least frequently studied age group being 12-year-old players (one study, 4%). A total sample of 5636 players were included across all studies examining aerobic capacity. Across all ages, studies most frequently assessed academy players (26 studies, 96%), followed by elite (eight studies, 30%), and amateur players (three studies, 11%). Seven studies (26%) reported data for players competing at multiple playing levels. Most studies (20 studies, 74%) did not report data according to playing position and grouped data for all players collectively.

**Table 5:** Aerobic capacity reported in male, adolescent rugby league players according to age group, playing level, and playing position

Study	Playing level	Position	Sample size (*n*)	Age (years)	Multistage Fitness Test predicted $\dot{\mathrm{V}}$O_2max_ (mL kg^−1^ min^−1^)	Yo-Yo Intermittent Recovery Test distance (m)
*12 years of age*						
Gabbett et al. [1]^a^	Academy	Forwards	13	12.5 ± 0.5	32.1 ± 4.1	–
	Academy	Backs	14	12.3 ± 0.5	36.2 ± 4.2	–
*13 years of age*						
Gabbett et al. [48]	Academy	All	53	13.2 ± 0.6	39.9 ± 6.9	
Gabbett et al. [1]^a^	Academy	Forwards	7	13.5 ± 0.5	40.5 ± 5.4	–
	Academy	Backs	10	13.7 ± 0.4	40.8 ± 7.4	–
Till et al. [51]	Academy	All	207	13.6 ± 0.3	47.2 ± 4.8	–
Till et al. [45]	Elite^b^	All	255	13.6 ± 0.3	46.4 ± 4.7	–
	Elite^c^	All	130	13.7 ± 0.2	49.5 ± 3.7	–
Till et al. [39]	Amateur	All	50	13.6 ± 0.3	45.1 ± 4.8	
	Academy	All	32	13.6 ± 0.2	47.3 ± 5.9	
	Elite	All	13	13.6 ± 0.3	47.0 ± 4.6	
Till et al. [8]	Amateur	All	249	13.6 ± 0.6	47.6 ± 5.6	–
	Academy	All	261	13.6 ± 0.6	49.6 ± 4.9	–
	Elite	All	70	13.8 ± 0.7	49.8 ± 4.6	–
Till et al. [50]	Amateur	All	NR	13.6 ± 0.2	54.5 ± 7.2	–
	Academy	All	NR	13.7 ± 0.1	47.7 ± 5.9	–
	Elite	All	NR	13.4 ± 0.3	48.6 ± 3.8	–
Till et al. [95]	Academy	All	NR	13.8 ± 0.1	48.6 ± 4.8	–
Till et al. [102]	Academy	All	NR	13.6 ± 0.2	47.9 ± 5.4	
		Outside backs	NR	13.7 ± 0.2	50.8 ± 3.8	
		Pivots	NR	13.5 ± 0.3	49.1 ± 3.7	
		Props	NR	13.6 ± 0.2	42.4 ± 7.2	
		Backs	NR	13.7 ± 0.2	47.4 ± 3.4	
*14 years of age*						
Gabbett et al. [47]	Academy	All	14	14.1 ± 0.2	43.3 ± 1.3	–
Gabbett et al. [1]^a^	Academy	Forwards	11	14.5 ± 0.5	38.5 ± 4.5	–
	Academy	Backs	12	14.6 ± 0.5	41.4 ± 4.6	–
Till et al. [51]	Academy	All	226	14.6 ± 0.3	48.7 ± 5.4	–
Till et al. [45]	Elite^b^	All	309	14.6 ± 0.3	48.7 ± 5.3	–
	Elite^c^	All	86	14.6 ± 0.3	50.9 ± 3.9	–
Till et al. [50]	Amateur	All	NR	14.6 ± 0.2	45.7 ± 5.4	–
	Academy	All	NR	14.7 ± 0.1	51.8 ± 4.5	–
	Elite	All	NR	14.4 ± 0.3	50.6 ± 3.7	–
Till et al. [102]	Academy	All	NR	14.6 ± 0.2	50.1 ± 4.7	
		Outside backs	NR	14.7 ± 0.2	51.8 ± 5.1	
		Pivots	NR	14.5 ± 0.3	50.1 ± 3.8	
		Props	NR	14.6 ± 0.2	46.2 ± 4.3	
		Backs	NR	14.7 ± 0.2	50.8 ± 4.0	
Till et al. [39]	Amateur	All	92	14.6 ± 0.3	47.0 ± 5.8	
	Academy	All	85	14.6 ± 0.3	49.1 ± 5.0	
	Elite	All	18	14.5 ± 0.3	49.3 ± 4.4	
Till et al. [95]	Academy	All	NR	14.8 ± 0.1	50.6 ± 5.0	–
Till et al. [59]	Academy	All	31	NR^f^	–	1027 ± 510
*15 years of age*						
Waldron et al. [46]	Academy	All	13	15.1 ± 0.3	48.1 ± 3.4	–
Dobbin et al. [66]	Academy	All	235	15.1 ± 0.8	–	727 ± 252
	Academy	Wingers		15.1 ± 0.8	–	756 ± 248
	Academy	Centres		15.1 ± 0.8	–	742 ± 252
	Academy	Halves		15.1 ± 0.9	–	808 ± 232
	Academy	Hookers		15.1 ± 0.1	–	777 ± 335
	Academy	Props		15.1 ± 0.1	–	591 ± 249
	Academy	Back rowers		15.1 ± 0.1	–	702 ± 216
Gabbet et al. [48]	Academy	All	20	15.1 ± 0.6	41.5 ± 7.2	–
Waldron et al. [52]	Academy	All	21	15.1 ± 0.3	47.0 ± 2.1	
Gabbett et al. [1]^a^	Academy	Forwards	12	15.4 ± 0.9	42.9 ± 4.4	–
	Academy	Backs	9	15.4 ± 0.5	49.5 ± 4.0	–
Till et al. [51]	Academy	All	204	15.5 ± 0.3	50.9 ± 4.6	–
Tredrea et al. [60]	Academy	All	49	15.5 ± 0.6	44.8 ± 5.1	–
	Elite	All	51	15.5 ± 0.4	48.1 ± 5.2	–
Till et al. [45]	Elite^b^	All	306	15.6 ± 0.3	50.6 ± 4.8	–
	Elite^c^	All	86	15.6 ± 0.3	51.1 ± 3.6	–
Till et al. [102]	Academy	All	NR	15.6 ± 0.2	51.3 ± 4.6	
		Outside backs	NR	15.7 ± 0.2	51.8 ± 4.6	
		Pivots	NR	15.5 ± 0.3	52.3 ± 3.4	
		Props	NR	15.6 ± 0.2	48.0 ± 5.0	
		Backs	NR	15.7 ± 0.2	52.6 ± 4.1	
Till et al. [39]	Amateur	All	107	15.6 ± 0.3	49.5 ± 5.1	
	Academy	All	144	15.6 ± 0.3	51.2 ± 4.5	
	Elite	All	39	15.5 ± 0.3	51.9 ± 3.8	
Till et al. [50]	Amateur	All	NR	15.6 ± 0.2	47.9 ± 4.6	–
	Academy	All	NR	15.7 ± 0.1	52.2 ± 5.3	–
	Elite	All	NR	15.4 ± 0.3	53.7 ± 2.9	–
Till et al. [61]	Academy	All	35	15.7 ± 0.2	–	1372 ± 443
Till et al. [95]	Academy	All	NR	15.8 ± 0.1	50.6 ± 4.6	–
Gabbett et al. [14]^a^	Academy	All	36	15.9 ± 0.6	43.3 ± 5.4	–
	Academy	Hit-up forwards		15.8 ± 0.7	42.1 ± 6.3	–
	Academy	Adjustables		15.7 ± 0.4	44.6 ± 5.6	–
	Academy	Outside backs^e^		16.1 ± 0.7	43.4 ± 4.7	–
	Elite	All^e^	28	16.0 ± 0.2	48.2 ± 4.6	–
	Elite	Hit-up forwards		15.9 ± 0.4	48.9 ± 4.1	–
	Elite	Adjustables^e^		16.0 ± 0.2	48.1 ± 5.1	–
	Elite	Outside backs^e^		16.0 ± 0.2	47.5 ± 4.6	–
Till et al. [96]	Academy	All	64		47.3 ± 3.4	
		Forwards	37	NR^g^	47.1 ± 3.7	–
		Backs	27	NR^g^	47.5 ± 3.0	–
*16 years of age*						
Waldron et al. [52]	Academy	All	21	16.2 ± 0.3	48.1 ± 3.9	
Waldron et al. [46]	Academy	All	13	16.2 ± 0.3	48.3 ± 3.6	–
Gabbet et al. [48]	Academy	All	15	16.5 ± 0.3	43.9 ± 5.8	–
Till et al. [61]	Academy	All	44	16.7 ± 0.2	–	1475 ± 327
Gabbett et al. [47]	Academy	All	21	16.9 ± 0.3	43.4 ± 1.1	–
Gabbet et al. [49]	Academy	All	36	16.9 ± 0.6	46.3 ± 3.2	
Till et al. [96]	Academy	All	46	NR^h^	48.7 ± 2.8	–
		Forwards	27	NR^h^	48.9 ± 3.2	–
	Academy	Backs	19	NR^h^	48.5 ± 2.1	–
Till et al. [103]	Academy	All	37	NR^h^	–	1436 ± 336
	Elite	All	13	NR^h^	–	1553 ± 287
Till et al. [59]	Academy	All	75	NR^h^	–	1234 ± 408
*17 years of age*						
Waldron et al. [52]	Academy	All	15	17.0 ± 0.4	51.7 ± 3.8	
Waldron et al. [46]	Academy	All	13	17.0 ± 0.3	52.2 ± 3.5	–
Dobbin et al. [62]	Academy	All	50	17.1 ± 1.1	–	766 ± 232
Dobbin et al. [42]	Academy	All	16	17.2 ± 0.7		638 ± 192
Pearce et al. [72]	Academy	All	52	17.2 ± 0.5	–	909 ± 313
Till et al. [97]	Academy	All	16	17.3 ± 0.7	–	1320 ± 242
Dobbin et al. [65]	Academy	All	197	17.4 ± 1.0		847 ± 205
		Back Row		17.5 ± 0.8		760 ± 188
		Centre		17.2 ± 0.9		845 ± 216
		Forward		17.3 ± 0.5		657 ± 108
		Full back		17.4 ± 0.9		957 ± 162
		Half Back		17.3 ± 1.1		933 ± 280
		Hooker		17.2 ± 1.2		1127 ± 187
		Loose Forward	17.5 ± 0.8		853 ± 278	
		Prop		17.2 ± 0.9		665 ± 209
		Scrum Half		17.3 ± 1.4		954 ± 171
		Second Row		17.9 ± 1.4		826 ± 171
		Stand-Off		18.0 ± 0.6		749 ± 231
		Winger		17.4 ± 1.1		835 ± 264
Tredrea et al. [60]	Academy	All	41	17.4 ± 0.7	45.6 ± 4.9	–
	Elite	All	19	17.0 ± 0.7	47.9 ± 7.1	–
Dobbin et al. [66]^d^	Elite	All	365	17.5 ± 2.0	–	775 ± 233
	Elite	Wingers		17.5 ± 2.0	–	773 ± 241
	Elite	Centres		17.5 ± 2.0	–	799 ± 226
	Elite	Halves		17.5 ± 2.1	–	871 ± 206
	Elite	Hookers		17.5 ± 2.2	–	960 ± 256
	Elite	Props		17.5 ± 2.3	–	615 ± 147
	Elite	Back rowers		17.5 ± 2.4	–	769 ± 215
Gabbett et al. [22]^d^	Academy	Props	37	17.6 ± 2.4	42.2 ± 7.4	–
	Academy	Hookers	31	17.3 ± 1.1	46.9 ± 7.1	–
	Academy	Second rowers	36	17.2 ± 1.8	45.1 ± 6.7	–
	Academy	Locks^e^	11	16.5 ± 1.5	44.6 ± 6.0	–
	Academy	Halfbacks^e^	27	16.7 ± 1.5	50.5 ± 5.3	–
	Academy	Five eighths^e^	11	16.7 ± 1.6	48.3 ± 6.1	–
	Academy	Centres	27	17.0 ± 2.3	47.1 ± 6.7	–
	Academy	Wings	39	17.7 ± 2.2	45.7 ± 6.2	–
	Academy	Fullbacks	21	17.4 ± 2.0	47.8 ± 5.2	–
Till et al. [61]	Academy	All	34	17.7 ± 0.3		1408 ± 281
Gabbett et al. [1]^a^	Academy	Forwards	10	17.8 ± 0.8	43.9 ± 5.0	–
	Academy	Backs	12	17.3 ± 0.8	46.1 ± 6.0	–
Gabbett et al. [23]^a^	Academy	All	36	17.9 ± 0.4	43.7 ± 12.6	–
Till et al. [96]	Academy	All	55		48.9 ± 2.9	
		Forwards	31	NR^i^	48.8 ± 3.3	–
		Backs	24	NR^i^	49.1 ± 2.2	–
Till et al. [103]	Academy	All	41	NR^i^	–	1464 ± 354
	Elite	All	19	NR^i^	–	1535 ± 322
Till et al. [59]	Academy	All	64	NR^i^	–	1223 ± 328
*18 years of age*						
Till et al. [61]	Academy	All	16	18.7 ± 0.2	–	1353 ± 352
Pearce et al. [72]	Academy	All	53	18.9 ± 0.6	–	894 ± 369
Till et al. [96]	Academy	All	44		48.5 ± 2.9	
		Forwards	25	NR^j^	48.3 ± 3.2	–
	Academy	Backs	19	NR^j^	48.9 ± 2.7	–
Till et al. [103]	Academy	All	30	NR^j^	–	1475 ± 443
	Elite	All	19	NR^j^	–	1443 ± 259

*NR* not reported, – not tested, *All* players combined across all positions

^a^Reported 95% confidence intervals, which were converted to standard deviation according to the Cochrane Handbook for Systematic Reviews of Interventions Version 5.1.0

^b^Players classified as elite by definition used in this review but as ‘regional’ by the authors

^c^Players classified as elite by definition used in this review but as ‘national’ by the authors

^d^Total sample size (all positions combined) was reported rather than independently for each position

^e^Sample age reported across multiple years, and across various playing positions (data allocated according to weighted mean age)

^f^Age provided categorically as under 14 years instead of being reported as mean ± standard deviation

^g^Age provided categorically as under 15 years instead of being reported as mean ± standard deviation

^h^Age provided categorically as under 16 years instead of being reported as mean ± standard deviation

^i^Age provided categorically as under 17 years instead of being reported as mean ± standard deviation

^j^Age provided categorically as under 18 years instead of being reported as mean ± standard deviation

### Muscular Strength

Table 6 shows data for the included tests examining upper-body and lower-body muscular strength, most frequently measuring 1RM (kg) using bench press and back squat, respectively. A total of nine studies examined muscular strength in male, adolescent rugby league players aged between 14 and 19 years. Most studies examined 17-year-old players (seven studies, 78%), with the least frequently studied age group being 14-year-old players (one study, 11%). No included studies examined 12- or 13-year-old players. A total sample of 743 players were included across all studies examining muscular strength. Across all ages, studies most frequently assessed academy players (seven studies, 78%), followed by elite players (two studies, 22%), with one study (11%) examining amateur players. Most studies (seven studies, 78%) did not report data according to playing position and grouped data for all players collectively.

**Table 6:** Muscular strength reported in male, adolescent rugby league players according to age group, playing level, and playing position

Study	Playing level	Position	Sample size (*n*)	Age (years)	Bench press 1RM (kg)	Back squat 1RM (kg)	Prone row 1RM (kg)	Methods		
								Attempts	Rest (min)	Equipment
*14 years of age*										
Alonso-Aubin et al. [84]	Amateur	All	46	14.5 ± 1.3	46.98 ± 13.59	104.26 ± 30.84	–	NR	NR	Smith machine
*15 years of age*										
Fernandes et al. [86]	Academy	All	16	15.4 ± 0.5	82.2 ± 12.6	–	–	NR	3	NR
Baker et al. [105]	Academy	All	13	15.5 ± 0.5	85.0 ± 10.4	–	–	NR	NR	BB
Till et al. [61]	Academy	All	35	15.7 ± 0.2	74.8 ± 12.5	101.8 ± 18.8	72.2 ± 9.7	3	3	BB
Till et al. [96]	Academy	All	31, 30, 31^f^	NR^b^	73.9 ± 13.2	100.4 ± 21.9	70.9 ± 10.1	3	3	BB
		Forwards	16	NR^b^	76.8 ± 10.9	105.2 ± 17.3	72.6 ± 8.5			
		Backs	15	NR^b^	70.9 ± 15.0	94.9 ± 25.7	68.9 ± 11.6			
*16 years of age*										
Baker et al. [105]	Academy	All	11	16.2 ± 1.2	70.0 ± 7.4	–	–			BB
Till et al. [61]	Academy	All	44	16.7 ± 0.2	93.9 ± 13.4	123.6 ± 17.1	84.0 ± 10.6	3	3	BB
Till et al. [96]	Academy	All	48	NR^c^	93.3 ± 13.4	122.2 ± 18.7	83.5 ± 10.2			
		Forwards	28	NR^c^	96.0 ± 13.6	124.9 ± 18.8	86.3 ± 9.3	3	3	BB
		Backs	19	NR^c^	89.3 ± 12.6	118.1 ± 18.8	79.4 ± 10.5			
Till et al. [103]	Academy	All	37	NR^c^	92.1 ± 13.1	119.1 ± 19.5	81.8 ± 9.9	3	3	BB
	Elite	All	13	NR^c^	96.6 ± 14.4	131.0 ± 14.0	88.3 ± 10.3			
*17 years of age*										
Fernandes et al. [86]	Academy	All	23	17.1 ± 1.0	111.5 ± 14.3	–	–	NR	3	NR
Baker et al. [105]	Academy	All	15	17.1 ± 0.6	98.2 ± 13.5	–	–	NR	NR	BB
Till et al. [97]	Academy	All	61	17.3 ± 0.7	93.7 ± 16.7	115.7 ± 16.7	82.3 ± 11.7	3	3	BB
Till et al. [61]	Academy	All	34	17.7 ± 0.3	110.3 ± 15.9	138.2 ± 16.3	93.9 ± 11.0	3	3	BB
Till et al. [96]	Academy	All	55	NR^d^	103.7 ± 15.3	134.0 ± 15.5	91.1 ± 10.1	3	3	BB
		Forwards	31	NR^d^	107.5 ± 15.3	136.9 ± 14.2	94.3 ± 8.6			
		Backs	24	NR^d^	98.5 ± 13.8	129.6 ± 16.8	86.8 ± 10.4			
Till et al. [103]	Academy	All	41	NR^d^	100.8 ± 14.2	131.6 ± 14.2	89.6 ± 9.2	3	3	BB
	Elite	All	19	NR^d^	111.9 ± 15.7	139.6 ± 17.2	94.2 ± 11.1			
Till et al. [59]	Academy	All	64	NR^d^	92.6 ± 17.3	118.4 ± 23.8	82.0 ± 11.4	3	3	BB
*18 years of age*										
Till et al. [61]	Academy	All	16	18.7 ± 0.2	110.0 ± 15.3	134.0 ± 19.5	94.3 ± 11.8	3	3	BB
Kirkpatrick et al. [13]	Elite	Forwards	12	18.8 ± 1.1	101.7 ± 9.1	132.71 ± 9.4		3–5	2–4	BB
		Backs	12	18.7 ± 0.8	110.0 ± 15.8	140.21 ± 26.2				
Till et al. [96]	Academy	All	48	NR^e^	113.3 ± 16.4	138.4 ± 19.6	97.6 ± 2.4	3	3	BB
		Forwards	27	NR^e^	115.6 ± 16.3	143.7 ± 17.9	101.2 ± 11.4			
		Backs	21	NR^e^	110.0 ± 16.3	132.1 ± 20.2	93.0 ± 12.4			
Till et al. [103]	Academy	All	30	NR^e^	111.8 ± 15.4	135.7 ± 18.1	94.3 ± 11.5	3	3	BB
	Elite	All	19	NR^e^	115.6 ± 18.0	143.9 ± 20.1	102.8 ± 12.2			

*1RM* one-repetition maximum, *NR* not reported, – not tested, *All* players combined across all positions, *BB* Barbell

^a^Total sample size (all positions combined) was reported rather than independently for each position

^b^Age provided categorically as under 15 years instead of being reported as mean ± standard deviation

^c^Age provided categorically as under 16 years instead of being reported as mean ± standard deviation

^d^Age provided categorically as under 17 years instead of being reported as mean ± standard deviation

^e^Age provided categorically as under 18 years instead of being reported as mean ± standard deviation

^f^Sample size for each test varied so data are reported separately for Bench press 1RM (*n* = 31), Back squat 1RM (*n* = 30), Prone row 1RM (*n* = 31), 40-m (*n* = 178) in order

When examining the protocols used to assess 1RM, all studies (nine studies, 100%) clearly reported the range-of-motion required for each exercise. Five studies (56%) reported that three attempts were permitted to achieve 1RM, three studies (33%) did not report the number of attempts permitted, and one study (11%) reported three to five attempts were permitted. Six studies (67%) reported a 3-min rest between each attempt, two studies (22%) did not report the rest duration permitted between each attempt, and one study (11%) reported 2–4 min of rest was permitted between each attempt. Most studies (seven studies, 78%) reported use of a barbell to assess 1RM, with one study (11%) not specifying the equipment used, and one study (11%) reported use of a Smith machine to assess 1RM.

### Muscular Power

Table 7 shows data for the included tests examining lower-body and upper-body muscular power, most frequently measuring jump height (cm) using a CMJ test and distance thrown (m) using a MBT test, respectively. A total of 31 studies examined muscular power in male, adolescent rugby league players aged between 12 and 19 years. Most studies examined 15-year-old players (18 studies, 58%), with the least frequently studied age group being 12-year-old players (one study, 3%). A total sample of 5797 players were included across all studies examining muscular power. Across all ages, studies most frequently assessed academy players (29 studies, 94%), followed by elite (10 studies, 32%), and amateur players (three studies, 10%). Eight studies (26%) reported data for players competing at multiple playing levels. Most studies (23 studies, 74%) did not report data according to playing position and grouped data for all players collectively.

**Table 7:** Muscular power reported in male, adolescent rugby league players according to age group, playing level, and playing position

Study	Playing level	Position	Sample size (*n*)	Age (y)	CMJ height (cm)	MBT distance (m)	Methods CMJ			Methods MBT		
							Arm swing	Trials	Equipment	Position	Ball	Trials
*12 years of age*												
Gabbett et al. [1]^a^	Academy	Forwards	13	12.5 ± 0.5	28.2 ± 10.8	–	NR	2 (best)	Chalk	–	–	–
	Academy	Backs	14	12.3 ± 0.5	30.8 ± 4.5	–						
*13 years of age*												
Gabbett et al. [48]	Academy	All	53	13.2 ± 0.6	37.0 ± 6.2	–	NR	3 (best)	Yardstick	–	–	–
Gabbett et al. [1]^a^	Academy	Forwards	7	13.5 ± 0.5	33.1 ± 6.8	–	NR	2 (best)	Chalk	–	–	–
	Academy	Backs	10	13.7 ± 0.4	38.5 ± 8.1	–						
Till et al. [51]	Academy	All	209	13.6 ± 0.3	37.9 ± 5.2	5.2 ± 0.7	No	3 (best)	Tekei	Seated	2 kg	3 (best)
Till et al. [45]	Elite^b^	All	255	13.6 ± 0.3	38.2 ± 5.1	5.1 ± 0.7	No	3 (best)	Tekei	Seated	2 kg	3 (best)
	Elite^c^	All	130	13.7 ± 0.2	39.6 ± 5.0	5.4 ± 0.6						
Till et al. [8]	Amateur	All	249	13.6 ± 0.6	39.2 ± 4.9	5.7 ± 0.9	No	3 (best)	Tekei	Seated	2 kg	3 (best)
	Academy	All	261	13.6 ± 0.6	39.9 ± 5.6	5.7 ± 0.9						
	Elite	All	70	13.8 ± 0.7	41.2 ± 7.2	5.8 ± 1.0						
Till et al. [50]	Amateur	All	NR	13.6 ± 0.2	37.5 ± 4.5	5.4 ± 0.8	No	3 (NR)	Tekei	Seated	2 kg	3 (best)
	Academy	All		13.7 ± 0.1	38.6 ± 4.7	5.4 ± 0.5						
	Elite	All		13.4 ± 0.3	38.7 ± 4.3	5.3 ± 0.8						
Till et al. [102]	Academy	All	NR	13.6 ± 0.2	38.9 ± 5.0	5.4 ± 0.6	No	3 (best)	Tekei	Seated	2 kg	3 (best)
		Outside backs	NR	13.7 ± 0.2	42.4 ± 4.8	5.4 ± 0.5						
		Pivots	NR	13.5 ± 0.3	38.4 ± 4.5	5.0 ± 0.6						
		Props	NR	13.6 ± 0.2	35.9 ± 4.1	5.6 ± 0.8						
		Backs	NR	13.7 ± 0.2	37.2 ± 4.0	5.6 ± 0.5						
Till et al. [39]	Amateur	All	50	13.6 ± 0.3	41.4 ± 4.8	5.6 ± 1.1	No	3 (best)	Tekei	Seated	2 kg	3 (best)
	Academy	All	32	13.6 ± 0.2	40.9 ± 6.2	5.7 ± 1.2						
	Elite	All	13	13.6 ± 0.3	42.8 ± 7.9	5.7 ± 0.9						
Till et al. [95]	Academy	All	NR	13.8 ± 0.1	39.4 ± 5.3	5.4 ± 0.5	No	NR	NR	Seated	2 kg	NR
*14 years of age*												
Gabbett et al. [47]	Academy	All	14	14.1 ± 0.2	41.5 ± 1.4	–	No	NR	Yardstick	–	–	–
Gabbett et al. [1]^a^	Academy	Forwards	11	14.5 ± 0.5	34.7 ± 8.2	–	NR	2 (best)	Chalk	–	–	–
	Academy	Backs	12	14.6 ± 0.5	37.1 ± 4.4	–						
Gabbett et al. [24]	Academy	All	23	14.5 ± 0.5	35.9 ± 6.8	–	Yes	2 (best)	Yardstick	–	–	–
	Elite	All	36	14.3 ± 0.9	50.0 ± 5.6	–						
Till et al. [51]^f^	Academy	All	227, 226	14.6 ± 0.3	40.8 ± 5.0	5.8 ± 0.6	No	3 (best)	Tekei	Seated	2 kg	3 (best)
Till et al. [45]	Elite^b^	All	309	14.6 ± 0.3	40.3 ± 5.3	5.8 ± 0.7	No	3 (best)	Tekei	Seated	2 kg	3 (best)
	Elite^c^	All	86	14.6 ± 0.3	41.9 ± 5.1	6.0 ± 0.5						
Till et al. [102]	Academy	All	NR	14.6 ± 0.2	41.3 ± 4.4	5.9 ± 0.5	No	3 (best)	Tekei	Seated	2 kg	3 (best)
		Outside backs	NR	14.7 ± 0.2	43.8 ± 3.7	5.9 ± 0.5						
		Pivots	NR	14.5 ± 0.3	40.0 ± 5.4	5.6 ± 0.6						
		Props	NR	14.6 ± 0.2	39.1 ± 4.0	6.1 ± 0.6						
		Backs	NR	14.7 ± 0.2	41.1 ± 3.4	6.2 ± 0.4						
Till et al. [39]	Amateur	All	92	14.6 ± 0.3	39.0 ± 4.8	5.8 ± 0.8	No	3 (best)	Tekai	Seated	2 kg	3 (best)
	Academy	All	85	14.6 ± 0.3	40.5 ± 5.1	5.8 ± 0.8						
	Elite	All	18	14.5 ± 0.3	39.6 ± 6.6	5.0 ± 1.7						
Till et al. [50]	Amateur	All	NR	14.6 ± 0.2	39.3 ± 3.3	5.8 ± 0.8	No	3 (NR)	Tekei	Seated	2 kg	3 (best)
	Academy	All		14.7 ± 0.1	42.2 ± 4.2	5.9 ± 0.4						
	Elite	All		14.4 ± 0.3	41.3 ± 3.9	6.0 ± 0.6						
Till et al. [95]	Academy	All	NR	14.8 ± 0.1	41.8 ± 4.3	6.0 ± 0.5	No	NR	NR	Seated	2 kg	NR
Till et al. [59]	Academy	All	31	NR^g^	38.9 ± 6.4	–	No	3 (best)	Just Jump Mat	–	–	–
*15 years of age*												
Waldron et al. [46]	Academy	All	13	15.1 ± 0.8	47.0 ± 3.0	–	NR	3 (best)	Just Jump Mat	–	–	
Dobbin et al. [66]	Academy	All	235	15.1 ± 0.7	33.3 ± 6.8	6.3 ± 0.9	No	2 (best)	Just Jump Mat	Squatted	4 kg	2 (best)
	Academy	Wingers		15.1 ± 0.8	33.3 ± 6.7	6.4 ± 0.7						–
	Academy	Centres		15.1 ± 0.8	34.1 ± 6.8	6.1 ± 1.2						
	Academy	Halves		15.1 ± 0.9	34.0 ± 6.4	5.9 ± 0.8						
	Academy	Hookers		15.1 ± 0.1	34.6 ± 6.5	6.0 ± 0.8						
	Academy	Props		15.1 ± 0.1	30.1 ± 7.3	6.8 ± 0.8						
	Academy	Back rowers		15.1 ± 0.1	33.7 ± 6.9	6.4 ± 0.6						
Gabbett et al. [48]	Academy	All	20	15.1 ± 0.6	45.0 ± 6.6	–	NR	3 (best)	Yardstick	–	–	
Waldron et al. [52]	Academy	All	21	15.1 ± 0.3	45.5 ± 4.2		No	3 (best)	Just Jump Mat			
Gabbett et al. [1] ^a^	Academy	Forwards	12	15.4 ± 0.9	38.0 ± 5.7	–	NR	2 (best)	Chalk	–	–	
	Academy	Backs	9	15.6 ± 0.5	41.2 ± 4.6	–						
Tredrea et al. [60]	Academy	All	49	15.5 ± 0.6	49.3 ± 6.6	–	NR	3 (best)	Yardstick	–	–	–
	Elite	All	51	15.5 ± 0.5	51.0 ± 5.6	–						
Till et al. [51]	Academy	All	207	15.5 ± 0.3	42.5 ± 5.3	6.5 ± 0.7	No	3 (best)	Tekei	Seated	2 kg	3 (best)
Till et al. [102]	Academy	All	NR	15.6 ± 0.2	43.4 ± 5.1	6.5 ± 0.6	No	3 (best)	Tekei	Seated	2 kg	3 (best)
		Outside backs	NR	15.7 ± 0.2	46.1 ± 4.3	6.3 ± 0.6	–	–	–	–		
		Pivots	NR	15.5 ± 0.3	42.3 ± 5.7	6.2 ± 0.6	–	–	–	–		
		Props	NR	15.6 ± 0.2	40.3 ± 4.0	6.7 ± 0.7	–	–	–	–		
		Backs	NR	15.7 ± 0.2	43.7 ± 4.8	6.8 ± 0.4	–	–	–	–		
Gabbett et al. [24]	Academy	All	28	15.6 ± 0.6	40.3 ± 8.5	–	Yes	2 (best)	Yardstick	–	–	–
	Elite	All	21	15.8 ± 0.3	53.1 ± 7.9	–						
Till et al. [50]	Amateur	All	NR	15.6 ± 0.2	41.3 ± 3.9	6.4 ± 0.9	No	3 (NR)	Tekei	Seated	2 kg	3 (best)
	Academy	All		15.7 ± 0.1	43.5 ± 4.9	6.5 ± 0.5	–	–				
	Elite	All		15.4 ± 0.3	43.9 ± 5.4	6.7 ± 0.5	–	–				
Gabbett et al. [14]	Academy	All	36	15.6 ± 0.6	46.9 ± 6.8	–	NR	3 (best)	Yardstick	–	–	
	Academy	Hit-up forwards	15.8 ± 0.7	39.7 ± 2.9	–	–	–				–	
	Academy	Adjustables		15.7 ± 0.4	48.8 ± 6.1	–	–	–				–
	Academy	Outside backs	16.1 ± 0.7	50.0 ± 5.8	–	–	–					
	Elite	All	28	16.0 ± 0.2	51.6 ± 7.7	–	–	–				
	Elite	Hit-up forwards	15.9 ± 0.4	48.6 ± 5.1	–	–	–					
	Elite	Adjustables		16.0 ± 0.2	51.5 ± 8.5	–	–	–				
	Elite	Outside backs	16.0 ± 0.2	54.6 ± 8.5	–	–	–					
Till et al. [45]	Elite^b^	All	309	15.6 ± 0.3	42.5 ± 5.4	6.4 ± 0.7	No	3 (best)	Tekei	Seated	2 kg	3 (best)
	Elite^c^	All	86	15.6 ± 0.3	43.5 ± 5.0	6.5 ± 0.6						
Till et al. [39]	Amateur	All	107	15.6 ± 0.3	38.8 ± 4.8	5.8 ± 0.8	No	3 (best)	Tekai	Seated	2 kg	3 (best)
	Academy	All	144	15.6 ± 0.3	39.6 ± 5.5	5.7 ± 0.8						
	Elite	All	39	15.5 ± 0.3	41.3 ± 7.1	5.9 ± 1.0						
Till et al. [61]	Academy	All	35	15.7 ± 0.2	45.8 ± 5.5	–	No	3 (best)	Just Jump Mat	–	–	
Till et al. [95]	Academy	All	NR	15.8 ± 0.1	43.8 ± 5.2	6.5 ± 0.5	No	NR	NR	Seated	2 kg	NR
Gabbett et al. [38]	Academy	All	13	15.9 ± 0.6	46.7 ± 7.0		Yes	3 (best)	Yardstick	–	–	–
Till et al. [96]	Academy	All	67	NR^h^	45.7 ± 5.2	–	No	3 (best)	Just Jump Mat	–	–	–
		Forwards	37	NR^h^	43.8 ± 5.0	–				–	–	–
		Backs	30	NR^h^	45.7 ± 5.2	–						–
Till et al. [59]	Academy	All	75	NR^h^	44.2 ± 5.7	–	No	3 (best)	Just Jump Mat	–	–	–
*16 years of age*												
Gabbett et al. [38]	Academy	All	28	16.0 ± 0.2	51.6 ± 7.7		Yes	3 (best)	Yardstick	–	–	–
Waldron et al. [52]	Academy	All	21	16.2 ± 0.3	45.6 ± 5.8		No	3 (best)	Just Jump Mat	–	–	–
Waldron et al. [46]	Academy	All	13	16.2 ± 0.3	47.3 ± 4.9	–	NR	3 (best)	Just Jump Mat	–	–	–
Gabbett et al. [48]	Academy	All	15	16.5 ± 0.3	47.3 ± 7.8	–	NR	3 (best)	Yardstick	–	–	–
Coutts et al. [64]	Academy	All	21	16.6 ± 1.2	50.3 ± 6.9		Yes	3 (best)	Yardstick			
	Academy	All	21	16.8 ± 1.0	51.1 ± 6.6							
Till et al. [61]	Academy	All	44	16.7 ± 0.2	48.7 ± 2.8	–	No	3 (best)	Just Jump Mat	–	–	–
Gabbett et al. [47]	Academy	All	21	16.9 ± 0.3	45.8 ± 1.4	–	No	NR	Yardstick	–	–	–
Gabbet et al. [49]	Academy	All	36	16.9 ± 0.6	54.4 ± 7.1		Yes	2 (best)	Yardstick			
Gabbett et al. [24]	Academy	All	25	16.9 ± 0.3	42.3 ± 9.3	–	Yes	2 (best)	Yardstick	–	–	–
	Elite	All	18	16.8 ± 0.3	58.9 ± 7.7							
Till et al. [96]	Academy	All	50	NR^i^	49.1 ± 5.8	–	No	3 (best)	Just Jump Mat	–	–	–
		Forwards	27	NR^i^	48.0 ± 5.6	–						
		Backs	23	NR^i^	50.5 ± 6.0	–						
Till et al. [103]	Academy	All	37	NR^i^	48.8 ± 6.1	–	No	3 (best)	Just Jump Mat	–	–	–
	Elite	All	13	NR^i^	49.5 ± 4.9	–						
*17 years of age*												
Waldron et al. [52]	Academy	All	15	17.0 ± 0.4	47.0 ± 5.5		No	3 (best)	Just Jump Mat			
Waldron et al. [46]	Academy	All	13	17.0 ± 0.3	47.6 ± 5.5	–	NR	3 (best)	Just Jump Mat	–	–	–
Dobbin et al. [62]	Academy	All	50	17.1 ± 1.1	34.8 ± 4.8	6.4 ± 0.8	No	2 (best)	Just Jump Mat	Squatted	4 kg	2 (best)
Pearce et al. [72]	Academy	All	52	17.2 ± 0.5	58.5 ± 6.1	–	NR	3 (best)	Yardstick	–	–	–
Dobbin et al. [42]	Academy	All	16	17.2 ± 0.7	34.7 ± 5.9	6.8 ± 0.8	No	2 (best)	Just Jump Mat	Squatted	4 kg	2 (best)
Till et al. [97]	Academy	All	61	17.3 ± 0.7	48.3 ± 3.0	–	No	3 (best)	Just Jump Mat	–	–	–
Tredrea et al. [60]	Academy	All	41	17.4 ± 0.7	55.1 ± 6.2	–	NR	3 (best)	Yardstick	–	–	–
	Elite	All	10	17.0 ± 0.7	55.7 ± 6.4	–						
Dobbin et al. [65]	Academy	All	197	17.4 ± 0.4	37.2 ± 5.2	7.1 ± 0.7	NR	NR	Just Jump Mat	Squated	4 kg	2 (best)
		Back Row		17.5 ± 0.8	39.1 ± 6.9	7.3 ± 0.5						
		Centre		17.2 ± 0.9	37.4 ± 5.1	7.3 ± 0.8						
		Forward		17.3 ± 0.5	38.1 ± 6.2	7.4 ± 0.5						
		Full back		17.4 ± 0.9	43.1 ± 6.7	6.9 ± 0.9						
		Half Back		17.3 ± 1.1	39.6 ± 4.3	6.7 ± 0.7						
		Hooker		17.2 ± 1.2	35.1 ± 5.0	6.5 ± 0.6						
		Loose Forward	17.5 ± 0.8	35.1 ± 4.4	7.5 ± 0.7							
		Prop		17.2 ± 0.9	34.5 ± 4.4	7.4 ± 0.8						
		Scrum Half		17.3 ± 1.4	33.3 ± 3.5	6.0 ± 0.7						
		Second Row		17.9 ± 1.4	37.6 ± 3.5	7.3 ± 0.7						
		Stand-Off		18.0 ± 0.6	30.9 ± 6.3	7.3 ± 1.0						
		Winger		17.4 ± 1.1	42.5 ± 6.4	7.6 ± 0.9						
Dobbin et al. [66]	Elite	All	365	17.5 ± 2.0	38.1 ± 6.3	7.1 ± 0.8	No	2 (best)	Just Jump Mat	Squatted	4 kg	2 (best)
	Elite	Wingers		17.5 ± 2.0	41.9 ± 7.3	7.2 ± 0.9						
	Elite	Centres		17.5 ± 2.0	39.8 ± 5.8	7.3 ± 0.8						
	Elite	Halves		17.5 ± 2.1	38.3 ± 6.0	6.8 ± 0.8						
	Elite	Hookers		17.5 ± 2.2	38.7 ± 5.3	6.8 ± 0.8						
	Elite	Props		17.5 ± 2.3	34.2 ± 5.0	7.2 ± 0.8						
	Elite	Back rowers		17.5 ± 2.4	37.2 ± 5.3	7.3 ± 0.7						
Gabbett et al. [22]^a^	Academy	Props	37	17.6 ± 2.4	44.0 ± 7.2	–	No	2 (best)	Yardstick	–	–	–
	Academy	Hookers	31	17.3 ± 1.1	47.9 ± 11.3	–						
	Academy	Back rowers	36	17.2 ± 1.8	49.0 ± 10.5	–						
	Academy	Locks^e^	11	16.5 ± 1.5	45.2 ± 9.8	–						
	Academy	Halves^e^	27	16.7 ± 1.5	50.4 ± 11.6	–						
	Academy	Five eighths^e^	11	16.7 ± 1.6	48.5 ± 8.2	–						
	Academy	Centres	27	17.0 ± 2.3	50.4 ± 8.9	–						
	Academy	Wingers	39	17.7 ± 2.2	45.4 ± 8.6	–						
	Academy	Fullbacks	21	17.4 ± 2.0	42.8 ± 10.0	–						
Till et al. [61]	Academy	All	34	17.7 ± 0.3	51.2 ± 5.5	–	No	3 (best)	Just Jump Mat	–	–	–
Gabbett et al. [1]^a^	Academy	Forwards	10	17.8 ± 0.8	37.9 ± 6.7	–	NR	2 (best)	Chalk	–	–	–
	Academy	Backs	12	17.3 ± 0.8	40.0 ± 7.7	–						
Gabbett et al. [23]^a^	Academy	All	36	17.9 ± 0.4	54.8 ± 14.7	–	NR	2 (best)	Yardstick	–	–	–
Till et al. [96]	Academy	All	56	NR^j^	50.6 ± 5.7	–	No	3 (best)	Just Jump Mat	–	–	–
		Forwards	32	NR^j^	49.1 ± 5.2							
		Backs	24	NR^j^	52.6 ± 5.7	–						
Till et al. [103]	Academy	All	41	NR^i^	50.2 ± 5.8	–	No	3 (best)	Just Jump Mat	–	–	–
	Elite	All	19	NR^i^	51.8 ± 5.2	–						
Till et al. [59]	Academy	All	64	NR^j^	48.1 ± 5.6	–	No	3 (best)	Just Jump Mat	–	–	–
*18 years of age*												
Till et al. [61]	Academy	All	16	18.7 ± 0.2	50.3 ± 4.1	–	No	3 (best)	Just Jump Mat	–	–	–
Kirkpatrick et al. [13]	Elite	Forwards	12	18.8 ± 1.1	50.6 ± 7.1	–	No	3 (best)	Just Jump Mat			
		Backs	12	18.7 ± 0.8	50.6 ± 5.0	–						
Pearce et al. [72]	Academy	All	53	18.9 ± 0.6	58.0 ± 7.3	–	NR	3 (best)	Yardstick	–	–	–
Till et al. [96]	Academy	All	45		52.5 ± 5.5	–	No	3 (best)	Just Jump Mat	–	–	–
		Forwards	24	NR^k^	51.2 ± 4.4	–						
		Backs	21	NR^k^	54.0 ± 6.2	–						
Till et al. [103]	Academy	All	30	NR^k^	51.5 ± 5.2	–	No	3 (best)	Just Jump Mat	–	–	–
	Elite	All	19	NR^k^	53.3 ± 5.6	–						

*NR* not reported, – not tested, *All* players combined across all positions, *RM* repeated measurement (2 or 3 trials)

^a^Reported 95% confidence intervals, which were converted to standard deviation according to the Cochrane Handbook for Systematic Reviews of Interventions Version 5.1.0

^b^Players classified as elite by definition used in this review but as ‘regional’ by the authors

^c^Players classified as elite by definition used in this review but as ‘national’ by the authors

^d^Total sample size (all positions combined) was reported rather than independently for each position

^e^Sample age reported across multiple years, and across various playing positions (data allocated according to weighted mean age)

^f^Sample size for each test varied so data are reported separately for CMJ (*n* = 227) and MBT (*n* = 226)

^g^Age provided categorically as under 14 years instead of being reported as mean ± standard deviation

^h^Age provided categorically as under 15 years instead of being reported as mean ± standard deviation

^i^Age provided categorically as under 16 years instead of being reported as mean ± standard deviation

^j^Age provided categorically as under 17 years instead of being reported as mean ± standard deviation

^k^Age provided categorically as under 18 years instead of being reported as mean ± standard deviation

When examining the protocols used to assess muscular power, most studies (19 studies, 61%) examining CMJ height (cm) reported no arm swing was permitted, three studies (10%) reported arm swing was permitted, and nine studies (29%) did not report whether arm swing was permitted during jumps. Nineteen studies (61%) reported the best jump height across three trials, and one study (3%) reported three trials were used but not how the final reported jump height was determined. Eight studies (26%) reported the best jump height across two trials, and three studies (10%) did not report the number of jumps permitted or how final reported jump height was determined. Twelve studies (39%) used the Just Jump Mat, 11 studies (35%) used a yardstick device, six studies (19%) used the Takei Jump System, one study (3%) used chalk markings on a wall, and one study (3%) did not report the equipment used to measure CMJ height. Six studies (60%) examining MBT distance (m) reported players throwing the medicine ball from a seated position and four studies (40%) reported players throwing the medicine ball from a squatting position. Six studies (60%) reported the best throw distance across three trials, three studies (30%) reported the best throw distance across two trials, and one study (10%) did not report the number of trials permitted or how final reported throw distance was determined. Six studies (60%) reported using a 2-kg medicine ball and four studies (40%) report using a 4-kg medicine ball.

### Weighted Means for Physical Qualities

Table 8 shows the calculated weighted mean data for the included tests examining physical qualities in male, adolescent rugby league players.

**Table 8:** Weighted means and standard deviations for physical qualities in male, adolescent rugby league players according to age group and test

Quality and test	12 years of age	13 years of age	14 years of age	15 years of age	16 years of age	17 years of age	18 years of age
*Anthropometric quality*							
Height (cm)	*–*	171.58 ± 7.66	174.18 ± 6.26	176.61 ± 6.50	174.53 ± 5.44	179.34 ± 5.61	180.01 ± 5.30
Seated height (cm)	*–*	88.43 ± 4.24	88.55 ± 3.67	90.75 ± 3.24	–	91.83 ± 2.24	–
Body mass (kg)	50.68 ± 9.32^c^	65.42 ± 11.44	68.13 ± 10.98	75.36 ± 10.86	78.78 ± 9.95	84.89 ± 10.76	87.35 ± 9.91
Skinfold thickness (mm) using Σ4 sites	*–*	37.73 ± 15.40	38.72 ± 15.91	40.22 ± 15.54	36.81 ± 13.37	39.01 ± 12.50	37.44 ± 11.52
Skinfold thickness (mm) using Σ7 sites	–	–	73.3– ± 8.4–^b^	74.05 ± 22.13	73.45 ± 22.37	92.94 ± 35.92	–
*Linear speed*							
10-m sprint time (s)	–	1.94 ± 0.12	1.94 ± 0.12	1.85 ± 0.13	1.80 ± 0.06	1.81 ± 0.62	1.81 ± 0.07
20-m sprint time (s)	^–^	3.36 ± 0.17	3.37 ± 0.17	3.11 ± 0.16	3.11 ± 0.09	3.11 ± 0.10	3.10 ± 0.12
*Change-of-direction speed*							
505 Agility Test(s)^a^	–	2.52 ± 0.15	2.49 ± 0.15	2.50 ± 0.15	2.30 ± 0.13	–	–
L-run (s)	–	–	–	–		8.60 ± 0.40	8.7 ± 0.4
*Aerobic capacity*							
MSFT (predicted $\dot{\mathrm{V}}$O_2max_ in mL kg^−1^ min^−1^)	34.23 ± 4.16^c^	47.50 ± 4.97	48.47 ± 5.11	49.58 ± 4.58	47.26 ± 0.51	46.65 ± 6.17	48.50 ± 2.90^b^
Yo-Yo Intermittent Recovery Test Level I (m)	–	–	1027 ± 510^I^	810.6 ± 276.76	1365.51 ± 361.85	915.65 ± 247.58	1192.35 ± 367.80
*Muscular strength*							
Bench press 1RM^b^	–	–		74.38 ± 12.83	93.48 ± 13.41	99.84 ± 16.02	112.82 ± 16.25
Back squat 1RM^b^	–	–		99.62 ± 19.92	122.63 ± 17.98	126.83 ± 17.71	137.99 ± 19.27
Prone row 1RM^b^	–	–	–	71.59 ± 9.89	83.65 ± 10.26	87.35 ± 10.81	97.13 ± 7.80
*Muscular power*							
Medicine ball throw (m)	–	5.47 ± 0.82	5.81 ± 0.70	6.25 ± 0.77	–	7.01 ± 0.80	–

*MSFT* multistage fitness test

^a^Data reported are weighted means combined as right foot and unidentified

^b^One repetition maximum

^c^Data from a single study and therefore not weighted across multiple studies

– data not reported

## Discussion

This review identifies the most frequently used tests in the literature to assess physical qualities, while concurrently scrutinising testing protocols that provide context to assist with data interpretation and future testing practices. The most frequently used tests to assess physical qualities were: (1) body mass; (2) standing height to measure height; and (3) Σ4 sites to measure skinfold thickness; (4) 10-m sprint test to assess linear speed; (5) 505 Agility Test to assess COD speed; (6) MSFT test to assess aerobic capacity; (7) back squat and bench press 1RM tests to assess lower-body and upper-body muscular strength, respectively; and (8) CMJ and MBT to assess lower-body and upper-body muscular power, respectively. Unfortunately, insufficient data were reported for each playing level and omissions combined with inconsistencies in categorising players according to positional groups across studies precluded the ability to calculate weighted means for each quality according to playing level and position. However, for most included tests in this review, sufficient data were available to provide a weighted mean value for test data according to age group. Notably, these weighted means only include studies which reported the most frequently used protocols consistently across all methodological considerations to permit collation of comparable data. Furthermore, when discussing each of the most frequently used tests below, we will present some critique regarding their application in practice using a recent framework proposed for test selection [37]. Specifically, we will identify the extent to which the reliability and validity of each test were reported specifically for adolescent, male rugby league players among the included studies, and also whether outcomes from each test can be used to guide training prescription [37]. Moreover, we will identify areas in need of further investigation such as additional testing options and deficiencies in evidence, while providing recommendations on the key protocols to adopt if implementing each test in practice.

### Anthropometric Qualities

Our data show the most frequently used tests to assess anthropometric qualities are body mass using electronic scales, height measured as standing height using a stadiometer, and skinfold thickness measured as Σ4 sites using Harpenden callipers (Table 1). Indeed, measurement of height and body mass is standard practice, and prior work has demonstrated the importance of measuring these qualities in male, adult rugby league players. Specifically, data from male, semi-professional rugby league players (22.5 ± 4.9 years) demonstrated height is significantly correlated (*p* < 0.05) with play-the-ball ability (*r* =  − 0.62), performing skills under fatigue (*r* =  − 0.60), and passing ability (*r* =  − 0.51) when assessed using subjective coach ratings [11]. Similarly, body mass was shown to be significantly correlated (*p* < 0.05) with the ability to hit and spin out of tackles (*r* = 0.49), offload out of tackles (*r* = 0.47), passing ability (*r* =  − 0.42), and catching ability (*r* =  − 0.38) when determined using subjective coach ratings [11]. To date, only one study has conducted similar investigation in male, adolescent rugby league players (15.8 ± 0.5 years) [38], but did not find any significant (*p* > 0.05) relationships between tackling proficiencies and standing height (*r* =  − 0.17) or body mass (*r* =  − 0.21). Nonetheless, given the known relationship between these physical qualities and career attainment [39], measurement of height and body mass and comparisons to benchmark standards may be appropriate in adolescent players. However, greater body mass alone is not desired [30]; instead, greater body mass *coupled* with lower skinfold thickness may be more advantageous to rugby league players [40]. Despite no available data in male, adolescent rugby league players, skinfold thickness assessed via Σ7 sites was shown to be significantly correlated (*p* < 0.05) with minutes played (*r* =  − 0.32), tackle attempts (*r* =  − 0.36), completed tackles (*r* =  − 0.38), dominant tackles (*r* =  − 0.36), and tackling efficiency (*r* =  − 0.31) [41] in male, professional rugby league players (23.8 ± 3.8 years). These data demonstrate that players with lower skinfold thickness may spend more time on field and be more effective in executing tackles; however, these weak correlations should be interpreted with caution with further research required to determine the relationship of skinfold thickness to performance during match-play. Consequently, physical qualities appear to correlate with several critical subjective performance metrics, and the periodic measurement of these qualities is warranted to monitor and manage body mass and composition to enhance the prospective performance and career outcomes of male, adolescent rugby league players.

Generally, our data show that standing height and body mass increase with age among male, adolescent rugby league players (Tables 2, 8), which is consistent with previous reviews [3, 4]. Furthermore, our data demonstrate Σ4 skinfold thickness is typically stable across adolescence, and broad standard deviations may be indicative of variations in players across studies (Table 8). The variability in these data may be attributed to the seasonal phase in which testing was applied. In this regard, significant reductions in skinfold thickness (Σ7 sites) have been observed in male, academy rugby league players (17.2 ± 0.7 years) [42] after an early pre-season training intervention lasting 45 days. These data suggest that skinfold thickness depends on training phase and thus the timing of studies can have a considerable impact on this physical quality [43]. These findings highlight the need to better report the seasonal phase in which skinfold assessments are performed. Indeed, most physical qualities may change across seasonal phases, and we therefore recommend the use of periodic testing strategies to best manage and monitor long-term player development; but most critically, we recommend that future research report the seasonal phase to improve the specificity of data available. Nonetheless, the tests used to assess anthropometric qualities and testing protocols adopted were largely consistent in the literature. The most frequently used protocols to assess the Σ4 skinfold thickness were using Harpenden callipers at the biceps, triceps, subscapular, and supra-iliac sites. Given the most frequently used protocol to determine Σ4 sites aligns with those stipulated by The International Society for the Advancement of Kinanthropometry (ISAK) [44], we recommend they continue to be used to ensure consistency across studies while aligning with industry standards.

Across studies using the Σ4 skinfold site thickness assessments included in this review, 13 reported the reliability statistics (ICC = 0.95–0.99; [8, 14, 22, 23, 38, 42, 45–51] CV = 0.3–3.5% [14, 22, 23, 46]) and none reported the validity for this approach specifically in adolescent, male rugby league players, highlighting the need for more evidence in this population to better inform practitioners when selecting tests to assess skinfold thickness. Moreover, while the sum of skinfold thickness is recognised as a proxy measure for body composition, there are no benchmarks for optimal skinfold thickness in male, adolescent rugby league, nor specific guidance on whether a reduced number of sites is the most appropriate option for this assessment. In this regard, practitioners may consider examining specific player positions (Table 2) for guidance on skinfold thickness qualities, given greater skinfold thickness may be beneficial in some positions compared to others, particularly in those experiencing higher collision rates [22]. We speculate that the Σ4 skinfold sites is likely used most frequently due to its ease of implementation in practice (i.e., low number of sites for efficient measurement with accessible equipment). However, at present the ecological validity of skinfold thickness in male, adolescent rugby league players is unknown, and future research should investigate if there are any associations between skinfold thickness and match play metrics in this population, before further recommendations regarding skinfold assessments are made.

### Linear Speed

Our data show the most frequently used test to assess linear speed is the 10-m sprint (s). Indeed, linear speed is a vital quality for rugby league players, with Waldron and colleagues [52] showing a significant relationship (*p* < 0.05) between 10-m sprint force (product of body mass and acceleration) and successful ball carries across all age groups (i.e., 15.1 ± 0.3 years, Spearman’s *R* = 0.61; 16.2 ± 0.3 years, Spearman’s *R* = 0.69; 17.0 ± 0.4 years, Spearman’s *R* = 0.64) examined in male, elite adolescent rugby league players (16.0 ± 1.3 years). These data not only demonstrate the importance of 10-m sprint speed, but clearly demonstrate the interdependence of key physical qualities in determining rugby league performance. Separate data from male, professional rugby league players (23.6 ± 0.5 years) demonstrate that ~ 68% of sprints during matches occur across distances < 20 m [53]. As such, these data clearly outline the utility of assessing 10-m sprint performances in relation to match demands. However, Gabbett [53] reported ~ 10% of sprints were > 40 m in distance for outside backs compared to ~ 5% for props and adjustables in male, professional rugby league players (23.6 ± 0.5 years). Therefore, certain positional groups may benefit from monitoring sprint performance over longer distances, given the relevance to position-specific match demands. Despite 10-m sprint testing being commonly employed, and evidence of a significant correlation (Pearson’s *r* = 0.60, *p* < 0.001) between tackling ability and 10-m sprint times in male, academy, adolescent rugby league players (15.8 ± 0.5 years) [38], further data supporting the validity of this test are derived only from older, professional, male rugby league players (24 ± 3 years) [54]. Consequently, research is required to objectively examine the validity of linear sprint tests in male, adolescent rugby league players, that is to determine whether test performance translates to the movement mechanics and demands experienced during match-play.

In general, improvements (Tables 3, 8) in 10-m sprint times *appear* to occur throughout adolescence, between 12- and 18-year-old players. However, sprint times do not *appear* to improve on a year-to-year basis, but instead stabilise across 13- to 14-year-old players and 15- to 18-year-old players, with negligeable differences in sprint times (s) between age groups in later years. Indeed, the stability of 10-m sprint times in older adolescents may indicate the attainment of adequate 10-m sprint performance, at least when assessed using an independent sprinting task. For instance, mean sprint times across the 15- to 18-year age groups are comparable to those reported in male, senior, elite rugby league players (25 ± 3 years) (forwards: 2.08 ± 0.08 s and backs: 2.01 ± 0.10 s) [55]. Certainly, some variation in sprint times between studies examining players aged 16–18 years is evident by the large weighted standard deviations calculated in these age groups, which is likely due to the starting position that players attained prior to sprinting. For example, three studies stated the starting positions of players to be a “pre-determined*”* distance behind the start line [1, 22, 56], but no specified distance was provided (e.g., 0.5 m). Without this detail, it is unknown whether the starting positions of players in these studies was relatively close to the first light gate, which will create less momentum when initially triggering timing to increase sprint times compared to players starting further behind the first light gate [57]. Similarly, concerns are noted within the 16-year-old age group, with a single study [48] reporting slower sprint times compared to other studies in this age group whereby the starting distance behind the first light gate was also not reported. Collectively, these differences in testing protocols may have inflated sprint times in these older age groups. Given the most frequently used methods to determine 10-m linear sprint performance (i.e., commencing 0.5 m from the start line, best of three trials being used, and electronic timing gates) align with those stipulated by the National Strength and Conditioning Association (NSCA) [58], we recommend they be used in future linear speed assessments to ensure consistency in application while aligning with industry standards.

Across studies using the 10-m sprint included in this review, 20 reported the reliability statistics (ICC = 0.75–0.95 [1, 14, 22, 23, 38, 45, 47–49, 51, 56, 59–65]; CV = 1.3–4.5% [42, 56, 60–62, 65, 66]), while one [66] reported the validity of this test specifically for adolescent, male rugby league players. Moreover, while performance time taken from the 10-m sprint can be used to calculate sprint speed, it is unlikely that players will reach peak speed across this distance limiting the ability to prescribe training plans using these data. In this regard, practitioners may consider including sprints across longer distances for enhanced prescriptive utility as well as greater specificity to sprints performed during matches in some positions [53]. When assessing sprints across longer distances, we suggest adopting the previously recommended protocols for 10-m linear speed testing to improve consistency in the rugby league literature. Another important finding was that few studies combined player body mass measurements with sprint results to calculate sprint momentum, and no studies reported running momentum, which has been demonstrated to differentiate between age groups and playing positions in rugby union [67]; these factors should be considered in future research given the relevance of this variable to collision sports like rugby league [4, 68].

### Change of Direction Speed

Our data show the most frequently used test to assess COD speed was the 505 Agility Test. Indeed, reactive movement patterns stimulated by an opponent’s actions [10], appropriate positioning when executing technically sound tackles [8, 40], and movements associated with line breaks [69] require highly developed COD speed. Given, the importance of COD speed to perform critical movement patterns during match-play, it is understandable that 505 Agility Test times have been identified as a significant determinant (*p* < 0.001) of career progression in male, adolescent rugby league players (13.6 ± 0.6 years) [8]. Indeed, COD speed appears a critical component for player success in rugby league; however, research exploring the relationships between COD speed and in-match metrics is not well documented. Specifically, only one study [38] has examined the relationship between 505 Agility Test time and tackling proficiencies (*r* =  − 0.20, *p* > 0.05) assessed via subjective coach ratings in male, adolescent rugby league players (15.8 ± 0.5 years) without identifying significant relationships. Nonetheless, given the known relationship between COD speed and career attainment [8], measurement of this quality and comparisons to benchmark standards are likely appropriate in adolescent players. Consequently, future work is required to explore the ecological validity of the 505 Agility Test in male, adolescent rugby league players and objectively examine the relationships between COD speed and match metrics.

Typically, our data show improvement in COD speed with increasing age (Tables 4, 8); however, 505 Agility Test data are limited to few age groups (13–16 years) (Table 4), perhaps due to studies adopting multidirectional COD tests (i.e., the L-run test) in older players as demonstrated in our synthesis (Table 4). Our data appear consistent with findings from a previous review [4], with improvements in COD speed with increased age likely explained by development of musculoskeletal strength (Table 8) and coordination throughout adolescence and maturation [4]. Given the most frequently used methods to determine 505-Agility Test performance (i.e., best of three trials being used and electronic timing gates), align with those recommended for linear sprints and are logical to implement, we recommend they be used in future COD speed assessments to ensure consistency in application.

Across studies using the 505-Agility Test included in this review, eight reported the reliability statistics (ICC = 0.82–0.92 [14, 24, 38, 39, 45, 47, 48, 51]; none reported CV), while none reported the validity of this test specifically for adolescent, male rugby league players. Moreover, while performance time taken from the 505-Agility Test indicates COD speed, no assessment of agility (i.e., whole-body movement with change in velocity and/or direction in response to an external stimulus [70]) is provided, with a severe lack of evidence for this quality in male, adolescent rugby league players. Use of appropriate tests to assess agility is essential in future research examining this population given most changes in movement patterns occur in response to external stimuli (e.g., opponent, ball) during training and match scenarios [71]. Nevertheless, if assessment of COD speed is desired, the 505-Agility Test neglects the plethora of multidirectional movements performed in rugby league match-play. In this regard, practitioners may consider using the L-run test [22, 23, 49, 72] (Table 1) that contains multidirectional movement patterns.

### Aerobic Capacity

Our data show the most frequently used test to estimate aerobic capacity was the MSFT, which incrementally applies increased speeds to measure endurance running capacity for estimation of $\dot{\mathrm{V}}$O_2max_. Indeed, the intermittent nature of adolescent rugby league match-play [11] combined with the large running distances covered [9] and high energetic demands associated with collisions [73], require a high capacity for aerobic energy supply to cope with match demands. Furthermore, a well-developed aerobic capacity allows for more rapid recovery between high-intensity activity bouts and less accumulated fatigue across matches [74], which is important to maintain skill execution given it deteriorates with increased fatigue, as previously demonstrated in soccer players [75, 76]. In this way, male, professional rugby league players (22.5 ± 4.9 years) with greater estimated $\dot{\mathrm{V}}$O_2max_ determined via the MSFT (high = 56.8 ± 1.5 mL kg^−1^ min^−1^, low = 52.1 ± 1.9 mL kg^−1^ min^−1^) engaged in significantly more (*p* < 0.05; Cohen’s ES = 0.7) total collisions during matches [77]. Furthermore, male, semi-professional rugby league players (22.5 ± 4.9 years) with greater $\dot{\mathrm{V}}$O_2max_ are reported to play-the-ball faster following line engagements (*p* < 0.05) compared to players with lower aerobic capacities as determined via the MSFT [11]. These findings suggest that a greater aerobic capacity may facilitate quicker recovery following intermittent bouts of running and tackle contests. Although aerobic capacity is undoubtedly an essential physical quality in rugby league, research exploring relationships between aerobic capacity and match metrics is derived from male, senior rugby league players, and yet to be determined for adolescent rugby league players.

Our mean data show aerobic capacity remains relatively stable with marginal fluctuations across age groups (Table 8). Indeed, although the absolute capacity to consume oxygen may increase [78], $\dot{\mathrm{V}}$O_2max_ relative to body mass may remain relatively stable across adolescence [79], particularly when considering players are likely accruing lean muscle mass (Table 2). Except for 12-year-old players, the general consistency in estimated $\dot{\mathrm{V}}$O_2max_ may suggest that players have obtained adequate levels of aerobic fitness, at least when assessed as an independent running task, to successfully compete as adolescent, rugby league players. Although our synthesis of male, adolescent rugby league players showed aerobic capacities similar to those of semi-professional, rugby league players (47.5 vs. 54.3 mL kg^−1^ min^−1^, respectively) [1], future research should confirm the capacity of this test to differentiate between differing playing levels [80]. Given the most frequently used protocols to determine aerobic capacity with the MSFT were uniform and in line with those originally stipulated by Ramsbottom et al. [26], we recommend they continue to be followed if implementing the MSFT.

Across studies using the MSFT included in this review, 12 reported the reliability statistics (ICC = 0.90–0.92 [1, 14, 22, 23, 45–49, 51, 60]; CV = 3.7% [52]), while none reported the validity of this test specifically for adolescent, male rugby league players. While there is support for the MSFT in providing an estimate of $\dot{\mathrm{V}}$O_2max_ [26], outcomes taken from this test lack utility for training prescription. Moreover, included studies adopting this test were conducted 6–21 years ago, with many newer tests aimed at assessing aerobic capacity emerging in recent years. Consequently, more contemporary tests to assess aerobic capacity that have been less frequently adopted in adolescent, male rugby league players, such as the 30–15 Intermittent Fitness Test, may counter this practical limitation and provide data with strong support for their application for training prescription in team sports [81, 82].

### Muscular Strength

Our data show the most frequently used tests to assess maximal muscular strength were the 1RM bench press and 1RM back squat, for upper-body and lower-body muscular strength, respectively. Desired outcomes in match scenarios such as physically dominating the opposition [69], halting attacking players [12], and generating maximal force when colliding with the defensive line [83], require highly developed muscular strength. Despite the prevalence of 1RM tests among the literature, no study has examined the relationship between 1RM performance and match metrics in male, adolescent rugby league players. However, Johnston and colleagues [74] reported that male, elite, adolescent rugby league players (19.2 ± 0.7 years) with greater strength assessed using the 3RM squat test (high: 145 ± 17 kg; low: 119 ± 9 kg) covered more total running distances (*p* = 0.04; Cohen’s ES = 0.73), covered more distance at high speed (> 5.1 km h^−1^; *p* = 0.01), were involved in more collisions (*p* = 0.03), and completed more repeated high-intensity efforts (*p* = 0.02) during match-play than players with less strength [74]. These data demonstrate the importance of assessing muscular strength; however, further research is essential to elucidate relationships between strength assessed via 1RM bench press and 1RM back squat tests and match metrics in male, adolescent rugby league players.

In general, improvements in upper-body maximal muscular strength (via the 1RM bench press and prone row tests) were apparent across consecutive age groups that were examined for this quality (Table 8). Similarly, improvements in lower-body muscular strength are evident between 14- and 18-year-old players, but improvements in 1RM squat strength are not evident at each age interval. Indeed, back squat performance between 14- and 15-year-olds is the only instance a younger age group demonstrated higher mean performance data than that of the successive age group. However, this finding is likely attributed to a single study examining 1RM back squat strength in 14-year-old players [84] using a Smith machine as opposed to a free barbell. Research suggests a Smith machine fully supports and stabilises the barbell [85], which may have permitted heavier loads to be lifted in this study of 14-year-old players [49] compared to studies examining 15-year-old players using a free barbell. Given the most frequently used methods to determine 1RM testing (i.e., three attempts to achieve 1RM with 3-min rest periods permitted between attempts using a 20-kg barbell) align with those stipulated by the NSCA, we recommend they continue to be used in future assessments involving 1RM testing to ensure consistency in application while aligning with industry standards.

Across studies using 1RM testing included in this review, three reported the reliability statistics (ICC = 0.80–0.98 [64, 84, 86]; CV = 3.6% [86]) while none reported the validity of these tests specifically for adolescent, male rugby league players. Despite 1RM testing being the most frequently used assessment of muscular strength, the isometric midthigh pull (IMTP) has been adopted more recently as an assessment of whole-body strength and power in senior (*n* = 33, 25.3 ± 3.4 years) and adolescent (*n* = 23, 18.3 ± 1.4 years) male, rugby league players [87]. While the IMTP has fewer technical demands, which may benefit adolescent rugby league players who likely have accrued less training experience than their senior counterparts [87], 1RM tests typically replicate fundamental resistance training movements with the acquired data allowing precise prescription of resistance exercise loads at an individual level [37]. Certainly, the IMTP may be used to determine peak force and rate of force development, providing information about whether emphasis should be placed on strength- or power-based training interventions [88]; however, the equipment required for IMTP assessment make it cost-prohibitive in many adolescent rugby league environments. While application of the IMTP is well documented [88], no evidence demonstrates the utility of IMTP data for exercise prescription in adolescent rugby league players, suggesting further research is needed on this topic.

### Muscular Power

Our data show the most frequently used tests to assess muscular power were the CMJ and MBT tests, for lower-body and upper-body muscular power, respectively. Indeed, the application of instantaneous force in tackles [69] and high-velocity movement patterns [83] is dependent on the ability to produce high levels of muscular power. Jump height derived from the CMJ is often used as a proxy measure of lower-body muscular power [17]. Indeed, CMJ height (cm) has been demonstrated to significantly correlate (*η* = 0.44, *p* < 0.05) with beating an opposing player when assessed via subjective coach ratings in male, elite rugby league players (22.5 ± 4.9 years) [11]. However, to date, no work has evaluated the relationship between CMJ and match metrics in male, adolescent rugby league players. Furthermore, no research has evaluated whether the MBT test is related to any match metric in rugby league players at any age, despite this test being widely used in the rugby league literature. Nonetheless, significant correlations between upper-body muscular power measured using a plyometric push-up test and tackling ability (*r* = 0.65, *p* = 0.01) have been reported in male, semi-professional rugby league players (23.1 ± 3.6 years) [12]. The examination of similar relationships between the MBT test and match metrics is needed in future research.

In general, our data show increased CMJ height with age among adolescent players (Table 8). Certainly, typical growth during adolescence in conjunction with an increase in resistance training programming likely contribute to the accruement of lean muscle mass [89], greater absolute strength [79] and power [90], and increased neuromuscular function [91] to increase force application. However, CMJ data among 17-year-old players are substantially lower than other age groups, which may be explained by four studies from the same authorship group that report considerably lower jump height [42, 62, 65, 66]. These studies report use of a correction equation, stipulated in previous research [92, 93], to be used when utilising the Just Jump system. Despite the use of this recommended equation, these studies have produced substantially lower jump heights than other work examining similar cohorts. Importantly, we note greater variability in equipment selection with the CMJ compared to any other test, which will likely impact data. For instance, evidence suggests substantial differences in jump height can result when using electronically predicted height (jump mat systems) compared to manual height determination (Yardstick device) [15, 94]; however, these findings may be due to differences in protocols and thus jump techniques, whereby manual devices require a reaching action that induce greater jump height. Differing protocols for the MBT were adopted between studies conducted by two authorship groups, primarily led by Till and colleagues [8, 45, 50, 51, 95] (62.5%) and by Dobbin and colleagues [42, 62, 66] (36.5%). Nevertheless, differences in protocols across studies did not appear to affect the increased performance during the MBT with advancing age.

Across studies using the CMJ test included in this review, 21 reported the reliability statistics (ICC = 0.90–0.97 [1, 14, 22, 23, 38, 42, 45, 46, 48, 49, 51, 52, 59–62, 64–66, 96, 97]; CV = 1.1–5.9% [42, 46, 52, 59–62, 65, 66, 96] while one [66] reported the validity of this test specifically for adolescent, male rugby league players. Although the CMJ test has been well investigated, the commonly reported jump height data is not directly used for exercise prescription nor a direct representation of muscular power. Consequently, alternative data derived from the CMJ may hold greater prescriptive utility [98]. Further, minor variations in jump strategies and equipment selection can profoundly affect test data; for instance, equipment that predicts jump height based on flight time such as jump mats, can be manipulated by the jump strategies adopted by players [99]. To this end, the lack of methodological consistency in protocols across studies included in this review meant we were unable to provide recommendations on the most frequently adopted protocols or calculate a weighted mean, and therefore benchmark data, for the CMJ. Given these variations, it is important that appropriate procedures with equipment suited to the desired data be adopted (e.g., force plate or linear position transducer for force or velocity measurement); however, the high expense associated with some technologies may make them cost-prohibitive for many adolescent rugby league teams, which should be considered. Across studies using the MBT test included in this review, six reported the reliability statistics (ICC = 0.74–0.97 [45, 51, 62]; CV = 0.6–9% [42, 65, 66]), while one [66] reported the validity of this test specifically for adolescent, male rugby league players. Similar to the CMJ test, alternative tests that offer greater prescriptive utility warrant further investigation, given throw distance is not directly translatable to an exercise prescription. Nonetheless, in line with the approach adopted in many studies included in this review and that recommended for other tests, the best of three trials should be adopted for the CMJ and MBT tests with suitable equipment employed to gather data meeting the practical needs in light of the constraints faced.

## Limitations

Although our review provides the most comprehensive assessment of the tests used, testing protocols adopted, and data obtained for physical qualities in male, adolescent rugby league players, several limitations should be acknowledged. First, this review included only common physical qualities previously identified as key determinants of career success in male, adolescent rugby league players [4]. Other physical qualities may be useful to consider as the evidence base grows in this field, which may include but are not limited to muscular endurance [100], mobility, and agility [80]. Second, for consistency, the data synthesised in this review were obtained from the first testing occasion reported in any study (i.e., prior to intervention and repeated assessments across the season). As a result of this approach, data are reported irrespective of seasonal phase, which may add variability to the dataset. Therefore, we recommend future research investigate changes in physical qualities across seasonal phases to further advance the understanding of longitudinal development in male, adolescent rugby league players. Unfortunately, few studies explicitly report the seasonal phase that testing occurred, and therefore seasonal phase was inferred in some instances based on the month and location of studies. Given pre-season intervention influences physical test data [59, 101], future work should clearly report the seasonal phase in which testing occurred to enable comparisons of like data. Third, data were typically reported according to chronological age rather than maturity status across published studies on this topic, which also aligns with the typical approaches adopted to delineate levels within adolescent rugby league competitions. However, we acknowledge that maturity status varies across adolescence and may yield different trends than what we observed if categorised this way. Fourth, we collated and scrutinised only key protocol-related aspects of each test included in our review and acknowledge these do not encompass all aspects that may influence the acquired data. However, we identified key aspects for each test that require precise implementation and reporting to ensure consistency in test administration and to minimise variations in data reported across future studies. Finally, our weighted mean dataset is representative of players competing across all playing levels and positions regardless of the protocols adopted for each specific test. Although we acknowledge it is important to establish normative data according to playing level and playing position given their impact on match demands [53] as well as physical qualities among rugby league players, a lack of research attention given to amateur and elite players combined with omission of, and inconsistencies in assigning, positional groups across studies precluded these calculations.

## Conclusion

Our review has identified the most frequently used tests as body mass, standing height, and Σ4 skinfold sites to assess anthropometric qualities, while the 10-m sprint, 505 Agility Test, MSFT, 1RM back squat and bench press, and CMJ and MBT were the most frequently used tests to assess linear speed, COD speed, aerobic capacity, muscular strength, and muscular power qualities, respectively. Further, our review identified and scrutinised the protocols adopted across studies for each of these tests, enabling us to provide recommendations in accordance with industry standards for most tests when implemented in future practice. While the reliability of most tests was supported across studies included in this review, many studies that reported reliability statistics within methodology sections did so without explicit mention of the procedures undertaken to derive them. Furthermore, the validity assessments were lacking, highlighting the need for further research exploring various forms of validity for each test to better inform practitioners on their suitability for implementation [37]. In addition, we synthesised and stratified data for each test according to age group, providing novel benchmarks for all the most frequently used tests except the CMJ test, which could not be provided due to variability and inconsistency in testing protocols across studies. These data may be used as age-specific references for male, adolescent rugby league players and should be further updated according to playing level and positional groups as more data become available.

### Supplementary Information

**Additional file 1: Table A.** Search terms and strategy used to retrieve studies examining the physical qualities of adolescent rugby league players.

**Additional file 2: Table B.** Modified Downs and Black checklist used to assess the methodological quality of the included studies.

**Additional file 3: Table C.** Results of risk of bias and methodological quality assessment for the included studies.

## Data Availability

Data generated and analysed in this study are included in this published article and its supplementary information files.

## Abbreviations

COD: Change of direction
PRISMA: Preferred reporting items of systematic reviews and meta-analyses
1RM: 1 Repetition maximum
CMJ: Countermovement jump
MBT: Medicine ball throw
Σ4: Sum of four skinfolds
MSFT: Multistage fitness test
ISAK: The International Society for the Advancement of Kinanthropometry
$\dot{\mathrm{V}}$O_2max_: Maximum oxygen consumption
