# Supplementary material for: What Tests are Used to Assess the Physical Qualities of Male, Adolescent Rugby League Players? A Systematic Review of Testing Protocols and Reported Data Across Adolescent Age Groups
Source: Sports Med Open. 2023 Nov 10;9:106. doi: 10.1186/s40798-023-00650-z (PMC10638136; doi:10.1186/s40798-023-00650-z)
Supplement: Supplementary file 1 — Additional file 1: Table A. Search terms and strategy used to retrieve studies examining the physical qualities of adolescent rugby league players. [file 40798_2023_650_MOESM1_ESM.docx]

**Additional file 1: Table A:** Search terms and strategy used to retrieve studies examining the physical and physiological characteristics of adolescent rugby league players.

| Search terms in Medline, PubMed, WebofScience, Embase, and Scopus | | |
| --- | --- | --- |
| Search 1 | Search 2 | Search 3 |
| Rugby League | Adolescent OR Youth OR Collegiate OR Junior | Fitness testing OR physical characteristics OR Testing OR physical performance OR  physical qualities OR physical profile OR anthropometric OR body height OR body weight OR skinfold OR body composition OR body fat OR power OR countermovement jump OR vertical jump OR broad jump OR muscular strength OR muscular endurance OR acceleration OR speed OR sprint OR running OR agility OR change of direction OR fitness OR physical fitness OR aerobic capacity OR repeated-sprint ability OR anaerobic capacity |
