# Supplementary material for: What Tests are Used to Assess the Physical Qualities of Male, Adolescent Rugby League Players? A Systematic Review of Testing Protocols and Reported Data Across Adolescent Age Groups
Source: Sports Med Open. 2023 Nov 10;9:106. doi: 10.1186/s40798-023-00650-z (PMC10638136; doi:10.1186/s40798-023-00650-z)
Supplement: Supplementary file 2 — Additional file 2: Table B. Modified Downs and Black checklist used to assess the methodological quality of the included studies. [file 40798_2023_650_MOESM2_ESM.docx]

**Additional file 2: Table B.** Modified Downs and Black checklist used to assess the methodological quality of the included studies

| **Question number** | **Question** |
| --- | --- |
|  | *Reporting* |
| 1 | Is the hypothesis/aim/objective of the study clearly described? |
| 2 | Are the main outcomes of the study clearly described? |
| 3 | Are the characteristics of the patients included in the study clearly described? |
| 4 | Are the main findings of the study clearly described? |
| 5 | Does the study provide estimates of the random variability in the data for the main outcomes? |
| 6 | Have actual probability values been reported (e.g., 0.035 rather than <0.05) for the main outcomes except where the probability value is less than 0.001? |
|  | *External validity* |
| 7 | Were the subjects asked to participate in the study representative of the entire population from which they were recruited? |
| 8 | Were those subjects who were prepared to participate representative of the entire population from which they were recruited? |
|  | *Internal validity-bias* |
| 9^a^ | If any of the results of the study were based on ‘data dredging’ was this made clear? |
| 10 | Were the statistical tests used to assess the main outcomes appropriate? |
| 11 | Were the main outcome measures used accurate (valid and reliable)? |

*Note****:*** ^a^, question 9 was deemed not applicable
