# Supplementary material for: What Tests are Used to Assess the Physical Qualities of Male, Adolescent Rugby League Players? A Systematic Review of Testing Protocols and Reported Data Across Adolescent Age Groups
Source: Sports Med Open. 2023 Nov 10;9:106. doi: 10.1186/s40798-023-00650-z (PMC10638136; doi:10.1186/s40798-023-00650-z)
Supplement: Supplementary file 3 — Additional file 3: Table C. Results of risk of bias and methodological quality assessment for the included studies. [file 40798_2023_650_MOESM3_ESM.docx]

**Additional file 3: Table C**. Results of risk of bias and methodological quality assessment for the included studies.

| **Study** | **Downs and Black checklist question number** | | | | | | | | | | | | | | | |  | | **Total** | |  |
| --- | --- | --- | --- | --- | --- | --- | --- | --- | --- | --- | --- | --- | --- | --- | --- | --- | --- | --- | --- | --- | --- |
|  | ***Reporting*** | | | | | |  | | ***External validity*** | | |  | | ***Internal validity-bias*** | | |  | |  |  |  |
|  | *1* | *2* | *3* | *4* | *5* | *6* | |  | | *7* | *8* | |  | | *10* | *11* | |  | |  | |
| Gabbett [22] | 1 | 1 | 0 | 1 | 1 | 0 | |  | | 0 | 0 | |  | | 1 | 1 | |  | | **6** | |
| Till et.el. [66] | 1 | 1 | 1 | 1 | 1 | 1 | |  | | 0 | 0 | |  | | 1 | 1 | |  | | **8** | |
| Till et.el. [62] | 1 | 1 | 1 | 1 | 1 | 1 | |  | | 1 | 1 | |  | | 1 | 1 | |  | | **10** | |
| Alonsa-Aubin et al. [49] | 1 | 1 | 1 | 1 | 1 | 1 | |  | | 0 | 0 | |  | | 0 | 0 | |  | | **6** | |
| Fernandes et al. [80] | 1 | 1 | 1 | 1 | 1 | 0 | |  | | 0 | 0 | |  | | 1 | 1 | |  | | **7** | |
| Dobbin et al. [32] | 1 | 1 | 1 | 1 | 1 | 0 | |  | | 0 | 0 | |  | | 1 | 1 | |  | | **7** | |
| Gabbett [1] | 1 | 1 | 1 | 1 | 1 | 1 | |  | | 0 | 0 | |  | | 1 | 1 | |  | | **8** | |
| Tredrea et al. [70] | 1 | 1 | 1 | 1 | 1 | 1 | |  | | 1 | 1 | |  | | 1 | 1 | |  | | **10** | |
| Till et al. [63] | 1 | 1 | 1 | 1 | 1 | 0 | |  | | 1 | 1 | |  | | 1 | 1 | |  | | **9** | |
| Nicholson et al. [39] | 1 | 1 | 1 | 1 | 1 | 1 | |  | | 0 | 0 | |  | | 1 | 1 | |  | | **8** | |
| Waldron et al. [82] | 1 | 1 | 1 | 1 | 1 | 1 | |  | | 0 | 0 | |  | | 1 | 1 | |  | | **8** | |
| Till et al. [61] | 1 | 1 | 1 | 1 | 1 | 1 | |  | | 1 | 1 | |  | | 1 | 1 | |  | | **10** | |
| Baker [79] | 1 | 1 | 1 | 1 | 1 | 0 | |  | | 0 | 0 | |  | | 1 | 0 | |  | | **6** | |
| Kirkpatrick et al. [13] | 1 | 1 | 1 | 1 | 1 | 1 | |  | | 0 | 0 | |  | | 0 | 0 | |  | | **6** | |
| Dobbin et al. [55] | 1 | 1 | 1 | 1 | 1 | 0 | |  | | 0 | 0 | |  | | 1 | 1 | |  | | **7** | |
| Till et al. [75] | 1 | 1 | 1 | 1 | 1 | 1 | |  | | 0 | 0 | |  | | 1 | 1 | |  | | **8** | |
| Coutts et al. [73] | 1 | 1 | 1 | 1 | 1 | 0 | |  | | 0 | 0 | |  | | 1 | 1 | |  | | **7** | |
| Dobbin et al. [56] | 1 | 1 | 1 | 1 | 1 | 0 | |  | | 1 | 1 | |  | | 1 | 1 | |  | | **9** | |
| Gabbett [74] | 1 | 1 | 1 | 1 | 1 | 0 | |  | | 0 | 0 | |  | | 1 | 1 | |  | | **7** | |
| Till et al. [76] | 1 | 1 | 1 | 1 | 1 | 1 | |  | | 0 | 0 | |  | | 1 | 1 | |  | | **8** | |
| Till et al. [71] | 1 | 1 | 1 | 1 | 1 | 1 | |  | | 0 | 0 | |  | | 1 | 1 | |  | | **8** | |
| Dobbin [77] | 1 | 1 | 1 | 1 | 1 | 1 | |  | | 1 | 0 | |  | | 1 | 1 | |  | | **9** | |
| Gabbett et al. [69] | 1 | 1 | 1 | 1 | 1 | 1 | |  | | 0 | 0 | |  | | 1 | 1 | |  | | **8** | |
| Pearce et al. [81] | 1 | 1 | 1 | 1 | 1 | 1 | |  | | 0 | 0 | |  | | 1 | 1 | |  | | **8** | |
| Gabbett et al. [14] | 1 | 1 | 1 | 1 | 1 | 1 | |  | | 0 | 0 | |  | | 1 | 1 | |  | | **8** | |
| Gabbett [23] | 1 | 1 | 1 | 1 | 1 | 0 | |  | | 0 | 0 | |  | | 1 | 1 | |  | | **7** | |
| Gabbett [67] | 1 | 1 | 1 | 1 | 1 | 0 | |  | | 1 | 1 | |  | | 1 | 1 | |  | | **9** | |
| Gabbett et al. [27] | 1 | 1 | 1 | 1 | 0 | 0 | |  | | 0 | 0 | |  | | 1 | 1 | |  | | **6** | |
| Darrell jones et al. [78] | 1 | 1 | 1 | 1 | 1 | 0 | |  | | 0 | 0 | |  | | 1 | 1 | |  | | **7** | |
| Waldron et al. [35] | 1 | 1 | 1 | 1 | 1 | 1 | |  | | 0 | 0 | |  | | 1 | 1 | |  | | **8** | |
| Dobbin et al. [57] | 1 | 1 | 1 | 1 | 1 | 0 | |  | | 0 | 0 | |  | | 1 | 0 | |  | | **6** | |
| Till et al. [8] | 1 | 1 | 1 | 1 | 1 | 0 | |  | | 1 | 1 | |  | | 1 | 0 | |  | | **8** | |
| Till et al. [64] | 1 | 1 | 1 | 1 | 1 | 1 | |  | | 0 | 0 | |  | | 1 | 0 | |  | | **7** | |
| Till et al. [66] | 1 | 1 | 1 | 1 | 1 | 1 | |  | | 0 | 0 | |  | | 1 | 1 | |  | | **8** | |
| Till et al. [68] | 1 | 1 | 1 | 1 | 1 | 0 | |  | | 0 | 0 | |  | | 0 | 1 | |  | | **6** | |
| Till et al. [28] | 1 | 1 | 1 | 1 | 0 | 1 | |  | | 1 | 1 | |  | | 1 | 1 | |  | | **9** | |
| Gabbett [24] | 1 | 1 | 1 | 1 | 1 | 0 | |  | | 0 | 0 | |  | | 1 | 1 | |  | | **7** | |

*Notes*: 1 = yes; 0 = no/unable to determine; the following questions were addressed: 1) Is the hypothesis/aim/objective of the study clearly described? 2) Are the main outcomes of the study clearly described? 3) Are the characteristics of the patients included in the study clearly described? 4) Are the main findings of the study clearly described? 5) Does the study provide estimates of the random variability in the data for the main outcomes? 6) Have actual probability values been reported (e.g., 0.035 rather than <0.05) for the main outcomes except where the probability value is less than 0.001? 7) Were the subjects asked to participate in the study representative of the entire population from which they were recruited? 8) Were those subjects who were prepared to participate representative of the entire population from which they were recruited? 9) If any of the results of the study were based on ‘data dredging’ was this made clear? 10) Were the statistical tests used to assess the main outcomes appropriate? 11) Were the main outcome measures used accurate (valid and reliable)?
